# Cognitive Resilience Training to Prevent PTSD and Major Depressive Disorder in Paramedic Recruits: A Randomized Clinical Trial

**DOI:** 10.1001/jamanetworkopen.2025.57241

**Published:** 2026-02-09

**Authors:** Jennifer Wild, Gabriella Tyson, Graham Thew, Abbie Wilkins, Esther Beierl, Shama El-Salahi, Hjördis Lorenz, Ceri Storch, Haddi Browne, Daniel Morris, Ed Watkins, Anke Ehlers

**Affiliations:** 1 Department of Experimental Psychology, University of Oxford, Oxford, United Kingdom; 2 Oxford Health National Health Service Foundation Trust, Littlemore, Oxford, United Kingdom; 3 Sir Henry Wellcome Building for Mood Disorders Research, School of Psychology, Faculty of Health and Life Sciences, University of Exeter, Exeter, United Kingdom

## Abstract

**Importance:**

Rates of posttraumatic stress disorder (PTSD) and major depressive disorder (MDD) are high among paramedics.

**Objective:**

To evaluate the efficacy of a cognitive resilience training program for reducing the development of PTSD and MDD among early career paramedics compared with psychoeducation and standard practice.

**Design, Setting, and Participants:**

This randomized clinical trial of paramedics training at 15 universities across England was conducted between October 2017 to October 2022 with 12-month follow-up. Data were analyzed from December 2023 to July 2025.

**Intervention:**

Participants were randomized to receive internet-delivered cognitive training in resilience (iCT-R), psychoeducation, or standard practice. iCT-R, a guided online intervention that utilizes cognitive therapy tools to target predictors of PTSD and MDD identified in prospective research with paramedics, consisted of 6 modules delivered over 6 weeks with 6-monthly top-up sessions delivered by email. Internet-delivered psychoeducation, a supported online psychoeducation intervention, consisted of 6 topics (1 topic per week) with 6-monthly top-up sessions delivered by email. Standard practice was training as usual.

**Main Outcomes and Measures:**

The primary outcome was rate of PTSD and MDD at 1-year follow-up, assessed by independent assessors blinded to intervention using the Structured Clinical Interview for the *Diagnostic and Statistical Manual of Mental Disorders* (*Fifth Edition*). Secondary outcomes included measures of PTSD and depression symptom severity, resilience, rumination, anxiety, psychological distress, and well-being. Intent-to-treat analyses were conducted, with the primary outcome analyzed using mixed-effects logistic regression.

**Results:**

Of 570 student paramedics enrolled (372 female [65.3%]; mean [SD] age, 23.67 [6.88] years), 195 were randomized to iCT-R, 197 to psychoeducation, and 178 to standard practice. For participants randomized to iCT-R, the odds of meeting criteria for PTSD or MDD at 12 months were significantly lower compared with psychoeducation (odds ratio [OR], 0.20; 95% CI, 0.05-0.73) and standard practice (OR, 0.25; 95% CI, 0.07-0.97). Providing iCT-R training to 18 to 24 paramedic trainees (number needed to treat) would prevent 1 case of PTSD or MDD.

**Conclusions and Relevance:**

In this randomized clinical trial, participants receiving iCT-R were approximately 5 times less likely to develop PTSD or MDD at 1-year follow-up compared with psychoeducation and 4 times less likely when compared with standard practice. These findings suggest that iCT-R appears to decrease the likelihood of developing PTSD and MDD in early career paramedics.

**Trial Registration:**

isrctn.org Identifier: ISRCTN16493616

## Key Points

**Question:**

Is training that targets modifiable risk factors for posttraumatic stress disorder (PTSD) and major depressive disorder (MDD) in paramedics associated with lower rates of these disorders at 1-year follow-up compared with psychoeducation and standard practice?

**Findings:**

In this randomized clinical trial of 570 student paramedics, participants who received internet-delivered cognitive training in resilience (iCT-R) over 6 weeks had a statistically significant reduction in and significantly lower odds of developing PTSD and MDD diagnoses at follow-up.

**Meaning:**

These findings suggest that iCT-R reduces the likelihood of developing common psychiatric disorders associated with paramedic training and work.

## Teaser

This randomized clinical trial evaluates the efficacy of a cognitive resilience training program for reducing the development of posttraumatic stress disorder (PTSD) and major depressive disorder (MDD) compared with psychoeducation and standard practice among early career paramedics in England.

## Introduction

Paramedics and those in training routinely save lives while contending with substantial stressors, which can include irregular shift patterns, time-sensitive response targets, and exposure to potentially traumatic events. The cumulative effects of such stressors elevate their risk for psychiatric disorders compared with the general population, particularly posttraumatic stress disorder (PTSD)^1^ and depression.^2^

It remains unclear which interventions may offer protective benefits for this population. A comprehensive review of preincident interventions for first responders found no evidence supporting preemployment screening or psychoeducation programs, limited evidence for well-being and stress management approaches, and modest promise for operational and line manager training, although high-quality trials are needed.^3^ The limited efficacy of existing approaches is illustrated by research showing that resilience training effects in student paramedics were lost by 6- and 9-month follow-up.^4^ Existing pretrauma approaches may lack efficacy for preventing trauma-related psychopathology because they fail to target disorder-specific modifiable risk factors and rely on psychoeducation-based formats.^5^ Targeting modifiable risk factors through active training may reduce the risk of developing trauma-related disorders. This approach has demonstrated efficacy in preventing probable depression and anxiety by targeting rumination as a risk factor^6^ and may hold promise for preventing PTSD.

Prospective research of student paramedics has identified 2 cognitive processes, rumination and maladaptive resilience appraisals, that significantly increase the likelihood of developing PTSD and major depressive disorder (MDD) during the first 2 years of service, even after controlling for past psychiatric history and trauma exposure.^7^ This randomized clinical trial evaluates internet-delivered cognitive training in resilience (iCT-R), which targets modifiable risk factors for PTSD and MDD in student paramedics. We compared iCT-R with psychoeducation and standard practice, hypothesizing that iCT-R would reduce rates of PTSD and depression at 1-year follow-up.

## Methods

### Study Design, Setting, and Participants

This single-blind randomized clinical trial took place at 15 sites across England from October 16, 2017, to October 31, 2022, enrolling student paramedics who were randomly assigned to iCT-R, psychoeducation, or standard practice. Eligible sites were universities offering a bachelor’s degree in paramedic science. The study was approved by the University of Oxford Medical Sciences Interdivisional Research Ethics Committee and followed the Consolidated Standards of Reporting Trials (CONSORT) reporting guideline. The statistical analysis plan and detailed institutional review board protocol are provided in Supplement 1. The trial protocol has been published and is available online.^8^ Independent follow-up assessments were conducted at postintervention (6 weeks), 1 year, and 2 years. One of the initial large collaborating sites introduced a psychoeducation resilience intervention before study recruitment began, preventing recruitment at that location. This necessitated expansion to additional sites and, combined with the effects of the COVID-19 pandemic, required extension of recruitment and study end dates. Although the original study intended to examine salivary and plasma samples for C-reactive protein and cortisol, these were collected at baseline but could not be collected at follow-up time points due to COVID-19 restrictions and are therefore not reported.

Eligible participants were student paramedics aged 18 years or older in any year of their 3-year training program. Participants completed the Posttraumatic Checklist for the *Diagnostic and Statistical Manual of Mental Disorders* (*Fifth Edition* [*DSM-5*]) (PCL-5)^9^ and the 9-item Patient Health Questionnaire (PHQ-9)^10^ for eligibility, followed by a telephone evaluation if above clinical cutoff. Those requesting treatment were referred to local services. Eligible participants provided informed consent electronically when they completed baseline questionnaires and completed a clinical interview. Questionnaires included demographic information on age, gender, and race and ethnicity (Asian, Black [African, Caribbean, or another Black background], White British, White other [defined as White Irish, Eastern European, or another White background], multiethnic, other [defined as any race or ethnicity not otherwise specified], and prefer not to say); race and ethnicity were included to describe the demographic characteristics of the sample. Participants who met criteria for current PTSD or MDD and requested treatment were excluded and referred to local services. Randomization occurred after baseline assessment.

Assessors were blinded to treatment randomization; participants were instructed not to disclose their randomization during assessments. Assessments were recorded and rerated if randomization was inadvertently revealed. Intervention fidelity was assessed by an independent rater.

### Sample Size Calculation

Based on Topper et al,^11^ we estimated a 50% relative risk reduction for iCT-R vs psychoeducation (relative risk = 0.50), with an anticipated incidence in the control groups of 25.0% and 12.5% in the intervention group. This 25.0% baseline estimate was based on PTSD prevalence in university students (25.3%)^12^ and paramedics (22%).^13^ With 80% power and α = .05, this required 152 participants per group. Accounting for 3 groups and 20% attrition, the target sample size was 570 participants.

### Randomization

Participants were randomized on a 1:1:1 ratio by a computer-generated randomization schedule stratified by site, gender, and baseline PHQ-9 score (≥9 vs <9) and PCL-5 score (≥33 vs <33). The randomization sequence was generated and implemented independently by the Oxford Clinical Trials Research Unit. The randomization sequence remained concealed from all study personnel.

### Interventions

eAppendix 1 and eTable 1 in Supplement 2 describe each intervention using core details from the Template for Intervention Description and Replication checklist. eTable 2 in Supplement 2 describes the modules by study condition, detailing their primary focus and associated tools. Both interventions were supplemented with weekly email-based coaching from 2 trained psychology graduates (coaches; G. Tyson and H.L.) who reviewed participants’ modules and responded with personalized templated emails. The coaches programmed automated SMS reminders of key points and practice prompts during the intervention, and monthly follow-up exercises for 6 months postintervention. Weekly supervision was provided to monitor adherence to protocol.

#### iCT-R

iCT-R is a 6-module program delivered weekly over 6 weeks. The program adopts the active, experiential approach of internet-delivered cognitive therapy interventions, incorporating strategies from internet-delivered cognitive therapy for PTSD,^14^ internet-delivered cognitive therapy for social anxiety disorder,^15^ and rumination-focused cognitive therapy for depression.^16^

#### Psychoeducation

The comparison intervention consisted of 6 weekly online psychoeducation modules covering sleep, stress, depression, anger, mindfulness, and PTSD, respectively. Modules were adapted from Mind (a UK mental health charity).

#### Standard Practice

Participants in this condition received their usual university training and support services. These participants did not receive any additional online modules or remote assistance.

### Primary Outcome

In line with the statistical analysis plan (Supplement 1), the primary outcome was PTSD and MDD diagnoses at 1-year follow-up with 2-year outcomes to be analyzed and reported separately. The primary outcome was determined by the Structured Clinical Interview for the *DSM-5*,^17^ administered by an independent assessor (S.E., A.W., C.S., D.M.) blind to intervention condition.

### Secondary Outcomes

Secondary outcomes included measures of symptom severity, resilience, and rumination. PTSD symptom severity was measured with the PCL-5 (items range from 0 [not at all bothered] to 4 [extremely bothered]),^9^ a 20-item self-report measure based on *DSM-5* criteria for PTSD, and depression symptom severity was measured with the PHQ-9 (items range from 0 [not all] to 3 [nearly every day]).^10^ Resilience was measured with the 25-item Wagnild Resilience Scale (WRS; items range from 1 [strongly disagree] to 7 [strongly agree])^18^ and the 25-item Connor-Davidson Resilience Questionnaire (CD-RISC; items range from 0 [not at all true] to 4 [true nearly all the time]).^19^ The WRS measures the capacity to withstand life stressors and includes items specific to attending to emergencies, while the CD-RISC assesses confidence in handling challenges.

Rumination was assessed with the 5-item brooding subscale of the Ruminative Responses Scale, which measures repetitive negative thinking in response to depressed mood (items range from 1 [almost never] to 4 [almost always])^20^ and the Responses to Intrusions Questionnaire (RIQ; items range from 0 [never] to 4 [always]),^21^ which measures dwelling in response to intrusive memories. Anxiety was measured with the 7-item Generalized Anxiety Disorder scale (items range from 0 [not at all] to 3 [nearly every day]).^22^ Psychological distress was measured with the 12-item General Health Questionnaire (items range from 0 [not at all] to 3 [much more than usual]).^23^ Well-being was assessed with the Warwick Edinburgh Mental Well-Being Scale (1 [none of the time] to 5 [all of the time]).^24^ Tertiary outcomes are detailed in eAppendix 2 and eTable 3 in Supplement 2.

### Fidelity and Interrater Reliability

Intervention fidelity was assessed by randomly selecting 20% of emails from both intervention conditions and evaluating 2 criteria: whether the email was sent (yes or no) and whether the email content matched the templated email in the well-being coach manual (yes or no). Fidelity assessment demonstrated 100% adherence to intervention protocols. Interrater reliability of diagnostic outcomes was assessed at the primary end point (1-year follow-up) by having a second blinded rater independently reevaluate all positive diagnoses plus a random sample of negative cases (84 cases). Interrater reliability was excellent (Cohen κ = 0.95).

### Statistical Analysis

The primary outcome measure was analyzed using mixed-effects logistic regression. This approach has the advantage of including all available data, accounting for repeated measures, and implicitly accounting for data missing at random. Models included categorical fixed factors of time (postintervention and 1-year follow up), condition (iCT-R, psychoeducation, and standard practice), and the time by condition interaction. The interaction permits the estimation of differences between treatments at each time point. The stratification variables of site, gender, baseline PHQ-9 score, and baseline PCL-5 score were included as fixed covariates, along with the baseline score on the measure being analyzed. Participant was specified as a random effect to account for between-subject variation. Analysis of the continuous outcome measures used linear mixed effects models following the same specification. Because data for the PHQ-9 and PCL-5 were also collected at 6-month follow-up, this time point was included in these models. All analyses used the intention-to-treat sample unless specified. Additional models were examined including only participants from the 2 active treatments who (1) met the minimum compliance threshold (completed modules 1 and 2 or 6) for their assigned treatment, and (2) completed the core modules of their assigned intervention (iCT-R: modules 3 [if-then for rumination] and 4 [then vs now for unwanted memories]; psychoeducation: module 3 [depression] and module 6 [PTSD]). The logistic models used maximum likelihood estimation and linear models used restricted maximum likelihood estimation. Q-Q plots indicated that the normality of residuals assumption was met for all models. Standardized between-group effect sizes were calculated by dividing the adjusted group difference by the baseline SD.

Baseline clinical and demographic variables were examined as possible predictors of missingness on the primary outcome at 1 year follow-up. One variable (hospitalization) showed a significant predictive association; participants who reported at baseline being hospitalized in the past 6 months were more likely to have missing data. As a sensitivity analysis, this variable was included as an additional covariate in the primary analysis model. This did not alter the substantive results of the model.

Mediation analyses were performed to examine whether the observed symptom scores (PHQ-9 and PCL-5) at 1 year were mediated by process variable scores (RIQ rumination subscale and the WRS) at postintervention. These used the same approach as Freeman et al,^25^ following the Baron et al^26^ procedure but using linear mixed-effects models to account for repeated measures. Posttreatment scores on each process variable were examined as a potential mediator of the association of randomization (each condition vs standard practice) with the 1-year symptom scores. All models included baseline scores on the outcome and mediator, and gender as covariates, with a random effect of participant nested within site. All analyses were conducted from December 2023 to July 2025 using R version 4.3.2 (R Project for Statistical Computing). Linear mixed-effects models were fitted using the nlme package,^27^ and mixed-effects logistic regression used the lme4 package.^28^ Statistical significance was defined as a 2-sided *P* < .05.

## Results

Of the 570 student paramedics who met eligibility criteria (372 female [65.3%]; mean [SD] age, 23.67 [6.88] years), provided informed consent and completed baseline questionnaires, 195 were randomized to internet-delivered cognitive training in resilience, 197 were randomized to receive psychoeducation, and 178 were randomized to standard practice (Figure). Table 1 shows the demographic characteristics of the sample.

**Figure.**
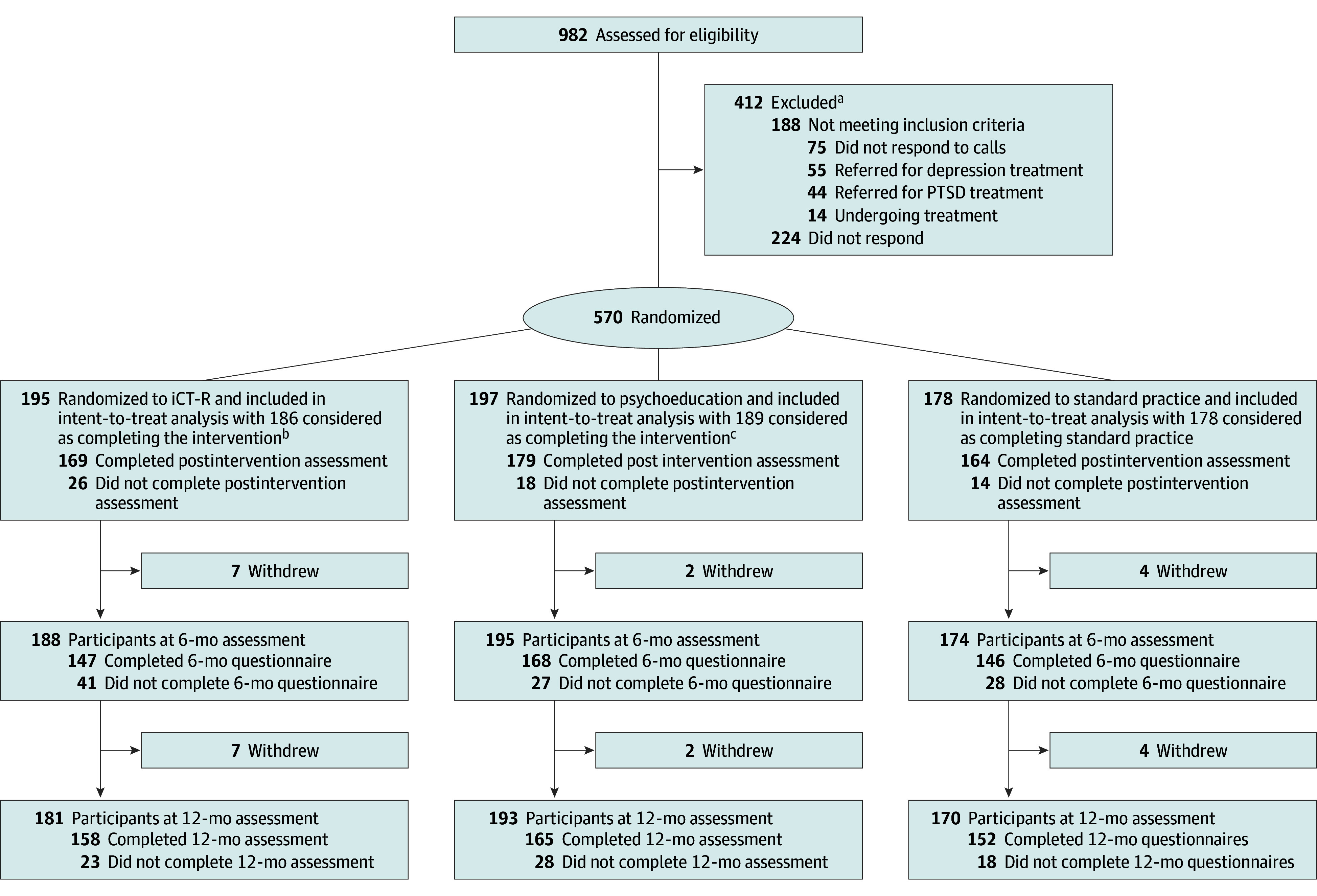
Flow Diagram PTSD indicates posttraumatic stress disorder. ^a^Thirty individuals were reincluded following screening. ^b^Of the 195 individuals randomized to iCT-R, 5 dropped out and 4 did not start the intervention. ^c^Of the 197 individuals randomized to psychoeducation, 8 dropped out.

**Table 1.** Baseline Demographic Characteristics of Randomized Participants^a^

Characteristic	Participants, No. (%)			
	iCT-R (n = 195)	Psychoeducation (n = 197)	Standard practice (n = 178)	Total (N = 570)
Gender				
Male	67 (34.4)	67 (34.0)	64 (36.0)	198 (34.7)
Female	128 (65.6)	130 (66.0)	114 (69.0)	372 (65.3)
Race and ethnicity				
Asian	5 (2.6)	4 (2.0)	7 (3.9)	16 (2.8)
Black (African, Caribbean, or another Black background)	1 (0.5)	3 (1.5)	4 (2.2)	8 (1.4)
Multiethnic background	8 (4.1)	4 (2.0)	5 (2.8)	17 (2.9)
White British	171 (87.7)	172 (87.3)	150 (84.3)	493 (86.5)
White (other)^a^	8 (4.1)	14 (7.1)	9 (5.0)	27 (4.7)
Other^b^	1 (0.5)	4 (2.0)	3 (1.7)	8 (1.4)
Prefer not to say	1 (0.5)	0	0	1 (0.2)
Age, mean (SD), y	23.37 (5.72)	23.90 (7.93)	23.76 (6.82)	23.67 (6.88)
Current relationship status				
Single	152 (77.9)	145 (73.1)	138 (77.5)	435 (76.3)
Long-term partner	30 (15.4)	32 (16.2)	22 (12.4)	84 (14.7)
Married	10 (5.1)	13 (6.6)	16 (9.0)	39 (6.8)
Divorced or separated	3 (1.5)	7 (3.6)	2 (1.1)	12 (2.1)
Education, mean (SD), y	15.88 (1.69)	15.82 (1.80)	15.92 (1.80)	15.87 (1.76)
Self-reported prior mental health diagnoses	30 (15.4)	35 (17.8)	32 (18.0)	97 (17.0)
Year of study				
1	108 (55.4)	107 (54.3)	109 (61.2)	324 (56.8)
2	59 (30.3)	59 (29.9)	44 (24.7)	162 (28.4)
3	28 (14.4)	31 (15.7)	25 (14.4)	84 (14.7)

Abbreviation: iCT-R, internet-delivered cognitive training in resilience.

^a^ White Irish, Eastern European, or another White background.

^b^ Any race or ethnicity not otherwise specified.

Participants demonstrated high compliance, with 142 participants (72.8%) completing all 6 modules in iCT-R, and 153 participants (77.6%) completing all 6 modules in psychoeducation. eTable 4 in Supplement 2 shows the rates of completion and compliance for both interventions and rates of participants achieving the minimum dose. eTable 5 in Supplement 2 shows rates of completion per module and the time spent on modules by intervention condition.

At 1-year follow-up, participants randomized to iCT-R were significantly less likely to meet PTSD or MDD criteria compared with standard practice (odds ratio [OR], 0.25; 95% CI, 0.07-0.97; *P* = .046) and psychoeducation (OR, 0.20; 95% CI, 0.05-0.73; *P* = .02). Providing iCT-R training to 18 to 24 paramedic trainees (number needed to treat) would prevent 1 case of PTSD or MDD. No significant differences in rates of diagnoses were found between psychoeducation and standard practice. Table 2 presents detailed comparisons of diagnostic outcomes and Table 3 shows comparisons for symptom severity scores at 1-year follow-up. Means and SDs of the tertiary outcomes at each time point are reported in eTable 3 in Supplement 2.

**Table 2.** iCT-R, Psychoeducation, and the Standard Practice Control Group and Diagnosis of PTSD or Major Depressive Disorder

Measure by time period	No. meeting criteria (%)			iCT-R vs standard practice		Psychoeducation vs standard practice		iCT-R vs psychoeducation	
	iCT-R (n = 195)	Psycho-education (n = 197)	Standard practice (n = 178)						
				Adjusted difference, OR (95% CI)	*P* value	Adjusted difference, OR (95% CI)	*P* value	Adjusted difference, OR (95% CI)	*P* value
SCID, PTSD, and/or depression									
Preintervention	2 (1.0)	1 (0.5)	0	1 [Reference]	NA	1 [Reference]	NA	1 [Reference]	NA
Postintervention	3 (1.5)	3 (1.5)	4 (2.2)	0.64 (0.13-3.03)	.57	0.59 (0.13-2.78)	.51	1.07 (0.21-5.58)	.93
12-mo follow-up	3 (1.5)	14 (7.1)	10 (5.6)	0.25 (0.07-0.97)	.046	1.27 (0.52-3.13)	.60	0.20 (0.05-0.73)	.02

Abbreviations: iCT-R, internet-delivered cognitive training in resilience; NA, not applicable; OR, odds ratio; PTSD, posttraumatic stress disorder; SCID, Structured Clinical Interview for the *Diagnostic and Statistical Manual of Mental Disorders* (*Fifth Edition*).

^a^ Mixed-effects logistic regression model including postintervention and 12-month time points, controlling for site, gender, baseline 9-item Patient Health Questionnaire score and baseline Posttraumatic Checklist for the *Diagnostic and Statistical Manual of Mental Disorders* (*Fifth Edition*) score. Baseline SCID status was not included in the model due to lack of convergence. Adjusted differences and confidence intervals given on the OR scale. There were no serious adverse events in any trial condition.

**Table 3.** Comparisons Between iCT-R, Psychoeducation, and Standard Practice on Continuous Outcome Measures^a^

Measure by time period	Unadjusted mean (SD) [No.]			iCT-R vs standard practice		Psychoeducation vs standard practice		iCT-R vs psychoeducation		Standardized between-group effect size (95% CI)		
	iCT-R	Psycho-education	Standard practice							iCT-R vs standard practice	Psychoeducation vs standard practice	iCT-R vs psychoeducation
				Adjusted difference (95% CI)	*P* value	Adjusted difference (95% CI)	*P* value	Adjusted difference (95% CI)	*P* value			
PCL-5												
Preintervention	7.17 (8.52) [195]	7.64 (8.60) [197]	6.84 (7.38) [178]	NA	NA	NA	NA	NA	NA	NA	NA	NA
Postintervention	4.65 (8.42) [169]	5.64 (8.32) [179]	5.54 (8.59) [164]	−1.13 (−2.93 to 0.66)	.22	−0.30 (−2.07 to 1.46)	.74	−0.83 (−2.58 to 0.92)	.35	0.14 (−0.08 to 0.36)	0.04 (−0.18 to 0.25)	0.10 (−0.11 to 0.31)
6-mo Follow-up	3.06 (4.62) [147]	4.40 (7.34) [168]	5.10 (7.54) [146]	−1.95 (−3.85 to −0.05)	.04	−1.04 (−2.88 to 0.80)	.27	−0.91 (−2.75 to 0.93)	.33	0.24 (0.01 to 0.47)	0.13 (−0.10 to 0.35)	0.11 (−0.11 to 0.34)
12-mo Follow-up	6.35 (8.63) [158]	8.79 (11.52) [165]	8.88 (12.80) [152]	−2.64 (−4.49 to −0.79)	.005	−0.59 (−2.42 to 1.24)	.53	−2.05 (−3.86 to −0.24)	.03	0.32 (0.10 to 0.55)	0.07 (−0.15 to 0.29)	0.25 (0.03 to 0.47)
PHQ-9												
Preintervention	3.66 (3.22) [195]	3.51 (3.16) [197]	3.14 (2.97) [178]	NA	NA	NA	NA	NA	NA	NA	NA	NA
Postintervention	3.06 (3.16) [169]	3.34 (3.80) [179]	3.76 (3.85) [164]	−0.85 (−1.61 to −0.09)	.03	−0.52 (−1.27 to 0.23)	.18	−0.33 (−1.07 to 0.41)	.38	0.27 (0.03 to 0.52)	0.17 (−0.07 to 0.41)	0.11 (−0.13 to 0.34)
6-mo Follow-up	2.79 (3.12) [148]	3.92 (4.17) [168]	3.70 (3.75) [146]	−0.96 (−1.76 to −0.16)	.02	0.15 (−0.63 to 0.93)	.70	−1.11 (−1.89 to −0.33)	.005	0.31 (0.05 to 0.56)	0.05 (−0.30 to 0.20)	0.36 (0.11 to 0.61)
12-mo Follow-up	3.36 (3.90) [158]	3.96 (4.16) [165]	4.27 (4.57) [152]	−1.03 (−1.82 to −0.25)	.01	−0.44 (−1.21 to 0.34)	.27	−0.59 (−1.36 to 0.17)	.13	0.33 (0.08 to 0.58)	0.14 (−0.11 to 0.39)	0.19 (−0.05 to 0.44)
GAD-7												
Preintervention	3.61 (3.71) [195]	4.32 (4.07) [197]	3.43 (3.48) [178]	NA	NA	NA	NA	NA	NA	NA	NA	NA
Postintervention	2.65 (3.46) [169]	2.93 (3.44) [179]	2.99 (3.39) [164]	−0.50 (−1.20 to 0.19)	.16	−0.45 (−1.14 to 0.24)	.20	−0.05 (−0.74 to 0.63)	.88	0.13 (−0.05 to 0.32)	0.12 (−0.06 to 0.30)	0.01 (−0.17 to 0.20)
12-mo Follow-up	2.84 (3.69) [158]	3.84 (4.03) [165]	3.23 (4.32) [152]	−0.45 (−1.17 to 0.27)	22	0.21 (−0.50 to 0.92)	.56	−0.66 (−1.37 to 0.04)	.07	0.12 (−0.07 to 0.31)	0.06 (−0.13 to 0.24)	0.18 (−0.01 to 0.36)
CD-RISC												
Preintervention	71.19 (10.62) [195]	71.48 (12.41) [197]	70.64 (12.08) [178]	NA	NA	NA	NA	NA	NA	NA	NA	NA
Postintervention	72.04 (11.51) [169]	72.83 (13.29) [179]	71.59 (13.40) [164]	0.35 (−1.67 to 2.37)	.73	0.12 (−1.88 to 2.11)	.91	0.24 (−1.74 to 2.21)	.81	0.03 (−0.14 to 0.20)	0.01 (−0.16 to 0.18)	0.02 (−0.15 to 0.19)
12-mo Follow-up	71.13 (12.48) [158]	71.36 (14.30) [165]	71.53 (13.01) [152]	−1.30 (−3.39 to 0.78)	.22	−1.81 (−3.87 to 0.26)	.09	0.50 (−1.54 to 2.55)	.63	0.11 (−0.07 to 0.29)	0.15 (−0.02 to 0.33)	0.04 (−0.13 to 0.22)
WRS												
Preintervention	135.10 (17.57) [195]	134.78 (19.27) [197]	130.88 (25.04) [178]	NA	NA	NA	NA	NA	NA	NA	NA	NA
Postintervention	132.68 (22.24) [169]	137.87 (19.12) [179]	134.39 (23.30) [164]	−3.55 (−7.91 to 0.80)	.11	1.63 (−2.66 to 5.92)	.46	−5.18 (−9.42 to −0.95)	.02	0.17 (−0.04 to 0.38)	0.08 (−0.13 to 0.28)	0.25 (0.05 to 0.45)
12-mo Follow-up	134.35 (21.98) [158]	134.70 (22.65) [165]	133.73 (23.77) [152]	−1.69 (−6.19 to 2.80)	.46	−1.34 (−5.79 to 3.11)	.56	−0.35 (−4.74 to 4.03)	.87	0.08 (−0.13 to 0.30)	0.06 (−0.15 to 0.28)	0.02 (−0.19 to 0.23)
WEMWBS												
Preintervention	50.63 (6.29) [195]	50.70 (7.43) [197]	50.40 (6.94) [178]	NA	NA	NA	NA	NA	NA	NA	NA	NA
Postintervention	51.56 (7.32) [169]	51.96 (7.68) [179]	50.48 (8.39) [164]	0.82 (−0.63 to 2.27)	.27	1.11 (−0.32 to 2.54)	.13	−0.29 (−1.70 to 1.13)	.69	0.12 (−0.09 to 0.33)	0.16 (−0.05 to 0.37)	0.04 (−0.16 to 0.25)
12-mo Follow-up	51.13 (8.22) [158]	50.39 (8.38) [165]	50.39 (8.70) [152]	0.34 (−1.15 to 1.84)	.65	−0.64 (−2.12 to 0.84)	.40	0.98 (−0.48 to 2.44)	.19	0.05 (−0.17 to 0.27)	0.09 (−0.12 to 0.31)	0.14 (−0.07 to 0.35)
ISI												
Preintervention	9.42 (4.45) [149]	9.28 (4.83) [150]	9.36 (4.74) [135]	NA	NA	NA	NA	NA	NA	NA	NA	NA
Postintervention	7.81 (4.34) [161]	8.39 (4.56) [174]	8.78 (4.92) [156]	−0.37 (−1.44 to 0.70)	.49	−0.29 (−1.34 to 0.76)	.59	−0.08 (−1.12 to 0.96)	.88	0.08 (−0.15 to 0.31)	0.06 (−0.16 to 0.29)	0.02 (−0.21 to 0.24)
12-mo Follow-up	8.32 (5.01) [158]	9.72 (5.28) [165]	9.28 (5.04) [152]	−0.59 (−1.68 to 0.50)	.29	0.34 (−0.74 to 1.41)	.54	−0.93 (−1.99 to 0.13)	.09	0.13 (−0.11 to 0.36)	0.07 (−0.16 to 0.30)	0.20 (−0.03 to 0.43)
GHQ												
Preintervention	22.35 (3.85) [195]	22.38 (4.09) [197]	22.02 (3.77) [178]	NA	NA	NA	NA	NA	NA	NA	NA	NA
Postintervention	21.26 (3.73) [169]	21.75 (4.35) [179]	22.48 (4.79) [164]	−1.33 (−2.30 to −0.35)	.008	−0.82 (−1.78 to 0.13)	.09	−0.50 (−1.45 to 0.45)	.30	0.34 (0.09 to 0.59)	0.21 (−0.03 to 0.46)	0.13 (−0.12 to 0.37)
12-mo Follow-up	22.13 (4.46) [158]	22.67 (5.31) [165]	23.51 (5.36) [152]	−1.48 (−2.48 to −0.48)	.004	−0.93 (−1.92 to 0.06)	.07	−0.55 (−1.53 to 0.43)	.27	0.38 (0.12 to 0.63)	0.24 (−0.02 to 0.49)	0.14 (−0.11 to 0.39)
RIQ: rumination subscale												
Preintervention	5.08 (4.66) [195]	5.64 (4.87) [197]	5.54 (4.36) [178]	NA	NA	NA	NA	NA	NA	NA	NA	NA
Postintervention	3.64 (3.97) [169]	4.18 (4.66) [179]	3.96 (4.05) [164]	−0.13 (−0.97 to 0.71)	.76	0.21 (−0.62 to 1.03)	.62	−0.34 (−1.16 to 0.48)	.42	0.03 (−0.15 to 0.21)	0.04 (−0.13 to 0.22)	0.07 (−0.10 to 0.25)
12-mo Follow-up	4.08 (4.96) [158]	4.35 (4.59) [165]	4.36 (4.11) [152]	−0.25 (−1.12 to 0.62)	.57	−0.18 (−1.04 to 0.68)	.68	−0.07 (−0.92 to 0.78)	.87	0.05 (−0.13 to 0.24)	0.04 (−0.15 to 0.22)	0.02 (−0.17 to 0.20)
RRS: brooding subscale												
Preintervention	8.98 (3.03) [195]	9.21 (3.42) [197]	8.98 (3.14) [178]	NA	NA	NA	NA	NA	NA	NA	NA	NA
Postintervention	7.88 (2.85) [169]	7.99 (3.14) [179]	7.68 (2.74) [164]	0.16 (−0.36 to 0.67)	.55	0.14 (−0.37 to 0.65)	.59	0.02 (−0.49 to 0.52)	.95	0.05 (−0.11 to 0.21)	0.04 (−0.12 to 0.20)	0.01 (−0.15 to 0.16)
12-mo Follow-up	8.75 (2.90) [158]	9.04 (2.85) [165]	8.82 (2.80) [152]	−0.09 (−0.63 to 0.44)	.73	0.07 (−0.45 to 0.60)	.78	−0.17 (−0.69 to 0.35)	.53	0.03 (−0.14 to 0.20)	0.02 (−0.14 to 0.19)	0.05 (−0.11 to 0.22)
Weight, kg												
Preintervention	73.71 (16.23) [193]	72.47 (15.43) [196]	73.97 (15.77) [177]	NA	NA	NA	NA	NA	NA	NA	NA	NA
Postintervention	74.19 (16.96) [167]	73.17 (15.74) [178]	74.51 (15.99) [161]	0.28 (−1.10 to 1.65)	.69	0.51 (−0.85 to 1.86)	.46	−0.23 (−1.57 to 1.11)	.74	0.02 (−0.07 to 0.10)	0.03 (−0.05 to 0.12)	0.01 (−0.07 to 0.10)
12-mo Follow-up	74.50 (16.25) [157]	73.87 (15.80) [163]	76.21 (17.42) [151]	−0.74 (−2.16 to 0.67)	.30	−0.21 (−1.61 to 1.19)	.77	−0.54 (−1.92 to 0.85)	.45	0.05 (−0.04 to 0.14)	0.01 (−0.08 to 0.10)	0.03 (−0.05 to 0.12)
Alcohol, unit^b^												
Preintervention	6.00 (9.14) [195]	5.36 (7.18) [197]	5.52 (8.44) [178]	NA	NA	NA	NA	NA	NA	NA	NA	NA
Postintervention	4.95 (6.54) [169]	5.06 (8.14) [179]	4.75 (8.61) [164]	−0.03 (−1.57 to 1.51)	.97	0.41 (−1.11 to 1.92)	.60	−0.44 (−1.94 to 1.06)	.57	<0.01 (−0.18 to 0.19)	0.05 (−0.13 to 0.23)	0.05 (−0.13 to 0.23)
12-mo follow-up	3.67 (5.31) [158]	5.67 (10.40) [165]	4.78 (7.39) [152]	−1.54 (−3.12 to 0.05)	.06	0.64 (−0.92 to 2.21)	.42	−2.18 (−3.73 to −0.63)	.006	0.19 (−0.01 to 0.38)	0.08 (−0.11 to 0.27)	0.26 (0.08 to 0.45)
Smoking, unit												
Preintervention	6.18 (6.16) [14]	9.81 (9.14) [21]	7.38 (5.99) [21]	NA	NA	NA	NA	NA	NA	NA	NA	NA
Postintervention	6.00 (5.62) [15]	7.80 (4.00) [15]	7.84 (5.76) [19]	0.31 (−2.25 to 2.87)	.81	−0.37 (−2.63 to 1.90)	.74	0.68 (−2.00 to 3.35)	.61	0.04 (−0.30 to 0.39)	0.05 (−0.26 to 0.36)	0.09 (−0.27 to 0.45)
12-mo follow-up	6.17 (6.06) [12]	9.94 (11.56) [17]	10.31 (5.92) [16]	−1.49 (−4.44 to 1.46)	.31	−3.41 (−6.09 to −0.73)	.02	1.92 (−0.83 to 4.67)	.17	0.20 (−0.20 to 0.60)	0.46 (0.10 to 0.82)	0.26 (−0.11 to 0.63)

Abbreviations: CD-RISC, Connor-Davidson Resilience Scale; GAD-7, Generalized Anxiety Disorder scale-7 item; GHQ, General Health Questionnaire; iCT-R, internet-delivered cognitive training in resilience; ISI, Insomnia Severity Index; PCL-5, Posttraumatic Checklist for the *Diagnostic and Statistical Manual of Mental Disorders* (*Fifth Edition*); NA, not applicable; PHQ-9, Patient Health Questionnaire-9 item; RIQ, Responses to Intrusions Questionnaire; RRS, Ruminative Responses Scale; WEMWBS, Warwick Edinburgh Mental Well-being Scale; WRS, Wagnild Resilience Scale.

^a^ Linear mixed-effects models including postintervention and 12-month time points, controlling for site, gender, baseline PHQ-9 score, and baseline PCL-5 score.

^b^ One-half of a pint of average strength beer or lager, 1 small glass of wine (76 mL), or 1 single measure of spirits, with visual examples provided.

Participants randomized to iCT-R showed significantly lower PTSD symptom severity at 1-year follow-up compared with psychoeducation (*d* = 0.25; 95%, CI, 0.03 to 0.47) and standard practice (*d* = 0.32; 95% CI, 0.10 to 0.55). For depression symptom severity, iCT-R participants demonstrated significantly lower severity compared with standard practice (*d *= 0.33; 95% CI, 0.08 to 0.58), and a small, nonsignificant advantage over psychoeducation (*d *= 0.19; 95% CI, −0.05 to 0.44). No significant differences in PTSD or depression symptom severity were found between psychoeducation and standard practice.

Participants rated the iCT-R and psychoeducation interventions comparably with no significant between-group differences for perceived course helpfulness (F_1,344_ = 0.433; *P* = .25) or email support (F_1,344_ = 1.72; *P* = .190). Both groups provided high ratings for course helpfulness (iCT-R: mean [SD], 74.28 [18.59]; psychoeducation: mean [SD], 75.65 [20.12]) with comparable ratings for well-being coach emails (iCT-R: mean [SD], 72.06 [23.54]; psychoeducation: mean [SD], 68.57 [25.80]). Participants who received iCT-R were significantly more likely to report practicing intervention tools compared with those who received the psychoeducation training at postintervention (χ^2^_1_ = 74.55; *P* < .001), 6-month follow-up (χ^2^_1_ = 57.71; *P* < .001), and 12-month follow-up (χ^2^_1_ = 40.23; *P* < .001).

Sensitivity analyses and complier average causal effect analyses are reported in eAppendix 3, eTable 6, and eTable 7 in Supplement 2, with sensitivity analyses not altering the substantive results. eTable 8 in Supplement 2 shows the results of mediation analyses. There were no significant indirect effects, indicating the absence of mediation by the 2 hypothesized predictors (ie, resilience appraisals and rumination).

## Discussion

This randomized clinical trial investigated the efficacy of iCT-R compared with psychoeducation and standard practice in a large sample of students training to be paramedics at university. Results demonstrated that student paramedics randomized to iCT-R had significantly lower rates of PTSD and MDD at 1-year follow-up compared with students randomized to psychoeducation and standard practice. Compared with psychoeducation, participants randomized to iCT-R demonstrated 5 times lower odds of meeting PTSD and/or MDD criteria at 12 months. Similarly, compared with standard practice, participants randomized to iCT-R demonstrated 4 times lower odds of meeting criteria for PTSD and/or MDD at 12-month follow-up.

Unlike psychoeducation-focused approaches, iCT-R offered an active, experiential intervention, teaching evidence-based tools through interactive exercises and videos. Participants had opportunities to apply and test strategies across modules, with the goal of targeting modifiable risk factors for PTSD and depression.

However, our mediation analyses did not support that the protective effects of the iCT-R intervention were mediated by questionnaires measuring rumination or resilience appraisals. This negative result likely stems from 2 key factors. First, there were floor effects because the sample comprised nonclinical participants at baseline. Correspondingly, they had, on average, very low scores in both the mediator and symptom measures at baseline and follow-up. Second, research demonstrates that many factors interact to lead to resilient outcomes, each exerting a modest effect.^29^ This suggests that protective mechanisms against PTSD development, such as low rumination, are more likely to exert modest rather than moderate to large effects and work in combination with other processes.

Our results are aligned with Bonanno et al^29^ in which resilience is observed to emerge from the flexible use of a toolkit of strategies adapted to individual circumstances. The iCT-R training equipped student paramedics with a repertoire of evidence-based tools drawn from cognitive therapy approaches, such as how to manage unwanted memories with stimulus discrimination,^30^ how to identify and reduce rumination,^16^ address excessive worry through realistic risk calculations,^30^ update negative self-beliefs^31^ associated with low resilience, shift self-focused attention,^32^ test fears of negative evaluation through behavioral experiments,^33^ and implement active strategies to target emerging PTSD and depression symptoms. Consistent with this active, experiential approach, participants who received iCT-R spent more time engaging with modules and were significantly more likely to report practicing tools compared with those receiving psychoeducation at all follow-up time points.

### Strengths and Limitations

Our study demonstrated several strengths in its design and evaluation, including recruiting a large sample of student paramedics, with long-term follow-up and inclusion of both active and comparator conditions. Both interventions benefited from monthly maintenance sessions for 6 months posttraining. Additionally, both interventions were designed as low-intensity protocols delivered by psychology graduates using templated email coaching under the supervision of a clinical psychologist, enhancing feasibility and potential scalability.

Our study has limitations. There were low rates of diagnoses at postintervention and follow-up across the groups, which limits the power to investigate predictors of outcome. Our predominantly White British sample reflects the demographic composition of UK paramedic students, limiting generalizability to more ethnically diverse populations. Our sample size may have been insufficient for mediation analyses, which require larger samples to detect indirect effects. We did not include an assessment to check the consolidation of learning for iCT-R or psychoeducation, or a comprehensive assessment of how much participants were implementing the tools they were learning in everyday life. However, since there were lower rates of PTSD and depression in iCT-R compared with psychoeducation and standard practice, we can be confident that the protective effects observed are intervention-specific. It would be of interest to determine whether effects persist at 2-year follow-up, which is currently being analyzed, as well as to explore whether modifying the intervention for trainees of high-risk occupations, such as firefighters and police officers, would show similar effects. This is currently under way in Australia.^34^

## Conclusions

The findings from this randomized clinical trial suggest that iCT-R appears to decrease the likelihood of developing PTSD and MDD in early career paramedics. iCT-R may offer a scalable approach to preventing trauma-related disorders in this high risk occupational group.

## Notes

Supplement 1. Institutional Review Board Protocol and Statistical Analysis Plan

Supplement 2. eAppendix 1. Description of Interventions eTable 1. Brief TiDiER Comparison Table: Internet-Delivered Cognitive Training in Resilience (iCT-R) vs Psychoeducation (Edu) eTable 2. Description of Modules by Condition eAppendix 2. Tertiary Outcomes eTable 3. Means and Standard Deviations or N (Percent) of Tertiary Outcome Measures at Each Time Point eTable 4. Rates of Participants Achieving Completion, Compliance and Minimum Dose Per Condition eTable 5. Rates of Completion Per Module and Time Spent on Modules by Intervention Condition eAppendix 3. Sensitivity, CACE and Mediation Analyses eTable 6. Sensitivity Analysis: Primary Analysis With the Three Baseline Clinical Cases Removed eTable 7. Sensitivity Analyses (Minimal Compliance and Core Module Completion): Odds of Meeting Criteria for PTSD and MDD at Follow-Up eTable 8. Mediation of 12-Month Scores on the PCL-5 and PHQ-9

Supplement 3. Data Sharing Statement
