# Supplementary material for: Cognitive Resilience Training to Prevent PTSD and Major Depressive Disorder in Paramedic Recruits: A Randomized Clinical Trial
Source: JAMA Netw Open. 2026 Feb 9;9(2):e2557241. doi: 10.1001/jamanetworkopen.2025.57241 (PMC12887744; doi:10.1001/jamanetworkopen.2025.57241)
Supplement: Supplement 1. — Institutional Review Board Protocol and Statistical Analysis Plan [file jamanetwopen-e2557241-s001.pdf]

Preventing PTSD, depression, and associated health problems in student paramedics: Protocol for a randomized controlled trial of internet-delivered cognitive training for resilience (iCT-R)

Short Title: PREVENT-PTSD
Ethics Ref: R44116/RE001
ISRCTN: ISRCTN16493616

**Principal Investigator:**	Dr Jennifer Wild, Department of Experimental Psychology, University of Oxford
**Investigators:**	Professor Anke Ehlers, Department of Experimental Psychology, University of Oxford
**Collaborators:**	Professor Carmine Pariante, NIHR Biomedical Research Centre, King's College London
	Professor Andrea Danese, Social, Genetic and Developmental Psychiatry, King's College London
	Dr. Apostolos Tsiachristas, Nuffield Department of Population Health, University of Oxford
**Research Staff:**	Gabriella Tyson, Department of Experimental Psychology, University of Oxford
	Shama El-Salahi, Department of Experimental Psychology, University of Oxford
	Hjordis Lorenz, Department of Experimental Psychology, University of Oxford

**Funder:**	MQ

Declarations of Conflict of Interest: Dr Jennifer Wild, Professor Anke Ehlers and their team have developed iCT-Resilience. They do not receive any income from this work.

Confidentiality Statement
This document contains confidential information that must not be disclosed to anyone other than the Sponsor, the Investigator Team, host organisation, and members of the Research Ethics Committee, unless authorised to do so.

TABLE OF CONTENTS

Chapter	Title	Page
1	Key Trial Contacts	3
2	Synopsis	5
3	Abstract	9
4	Background and Rationale	9
5	Objectives and Outcome Measures	11
6	Trial Design	13
7	Participant Identification	13
8	Trial Procedures	13
9	Interventions	15
10	Safety Reporting	16
11	Statistics	18
12	Data Management	20
13	Quality Assurance Procedures	20
14	Ethical and Regulatory Considerations	20
15	Finance and Insurance	21
16	Publication Policy	21
17	References	21
18	Appendix A: Trial Flow Chart	25
19	Appendix B: Schedule of Procedures	26
20	Appendix C: Amendment History	27

KEY TRIAL CONTACTS

**Principal Investigator**	Dr Jennifer Wild<br>Centre for Anxiety Disorders and Trauma<br>Department of Experimental Psychology<br>University of Oxford<br>The Old Rectory<br>Paradise Square<br>Oxford OX1 1TW<br>Email: jennifer.wild@psy.ox.ac.uk<br>TEL: 01865 618612
**Co-Investigator**	Professor Anke Ehlers<br>Centre for Anxiety Disorders and Trauma<br>Department of Experimental Psychology<br>University of Oxford<br>The Old Rectory<br>Paradise Square<br>Oxford OX1 1TW<br>Email: anke.ehlers@psy.ox.ac.uk<br>TEL: 01865 618600
**Clinical Trials Unit**	Oxford Clinical Trials Research Unit<br>Email: oxctrutrialshub@ndorms.ox.ac.uk<br>Tel: 01865 223469
**Statistician**	Dr Esther Beierl<br>Department of Experimental Psychology<br>University of Oxford<br>The Old Rectory<br>Paradise Square<br>Oxford OX1 1TW<br>Email: esther.beierl@psy.ox.ac.uk<br>Tel: 01865 618611
**Committees**	**Trial Steering Committee**<br><br>Dr Susan Dutton<br>Senior Medical Statistician and OCTRU Lead Statistician<br>Oxford Clinical Trials Research Unit<br>Centre for Statistics in Medicine<br>Nuffield Department of Orthopaedics, Rheumatology and Musculoskeletal Sciences<br>University of Oxford<br>Botnar Research Centre<br>Windmill Road<br>Oxford OX3 7LD<br>Email: susan.dutton@csm.ox.ac.uk<br>Tel: +44 (0) 1865 223451<br><br>Professor Willem Kuyken<br>Director, Oxford Mindfulness Centre<br>Department of Psychiatry<br>Warneford Hospital<br>Oxford OX3 7JX

	Email: willem.kuyken@psych.ox.ac.uk<br>Tel: +44 (0) 1865 618200<br><br>Dr Graham Harris<br>National Education Lead<br>College of Paramedics<br>Express Park, Bristol Rd<br>Bridgwater,<br>Somerset TA6 4RR<br>Email: graham.harris@collegeofparamedics.co.uk<br>Tel: +44 (0) 1278 420014<br><br>Professor Anke Ehlers<br>Centre for Anxiety Disorders and Trauma<br>Department of Experimental Psychology<br>University of Oxford<br>The Old Rectory<br>Paradise Square<br>Oxford OX1 1TW<br>Email: anke.ehlers@psy.ox.ac.uk<br>Tel: +44 (0) 1865 618600

SYNOPSIS

Trial Title	**Preventing PTSD, depression, and associated health problems in student paramedics: A randomized controlled trial of internet-delivered cognitive training for resilience**	
Internal ref. no. (or short title)	PREVENT-PTSD	
Trial Design	Randomized controlled trial	
Trial Participants	Students training to be paramedics at university in years 1, 2 or 3 of their university programme	
Planned Sample Size	570	
Training duration	6 weeks	
Follow up duration	6 months, 1 and 2 years	
Planned Trial Period	16 October 2017 – 30th January 2021	
	Objectives	Outcome Measures
Primary	**1**. Is internet-delivered cognitive training for resilience (iCT-R) more effective than accessing already available mental health information (Mind-Online) or having no specific training (standard practice), i.e., does iCT-R lead to fewer cases of PTSD and Major Depression (including subsyndromal PTSD and MD) and less PTSD and MD symptomatology at follow-up compared to Mind-Online and standard practice control conditions?	**1.**<br>Primary: Structured Clinical Interview for DSM-5 – PTSD and MD modules [24]<br><br>PTSD Symptom Checklist (PCL-5)[22]<br><br>Patient Health Questionnaire (PHQ-9)[23]

Secondary	**1.** Does internet-delivered cognitive training for resilience (iCT-R) lead to greater improvement in secondary outcome measures (resilience, rumination, hormone and immune function, smoking, weight gain, alcohol use, symptoms of anxiety, and sleep problems, psychological distress, wellbeing) than Mind-Online and standard practice conditions?<br><br>**2.** Is iCT-R more cost-effective than Mind-Online or standard practice, i.e., is the cost per participant without an episode or symptoms of PTSD or MD and costs per QALY gained smaller?	**1.**<br>Resilience: Connor Davidson Resilience Questionnaire (CD-RISC)[25]<br>Wagnild Resilience Scale[40]<br><br>Rumination: Ruminative Responses Scale (RRS)[37] (Brooding Subscale) & Responses to Intrusions Questionnaire (RIQ) dwelling subscale[33]<br><br>Anxiety: Generalized Anxiety Disorder 7-item Scale (GAD-7)[26]<br><br>Smoking & Alcohol Use (unpublished)<br><br>Weight & Height Questionnaire (unpublished)<br><br>Sleep problems: Insomnia Severity Index (ISI)[27]<br><br>Psychological distress: General Health Questionnaire (GHQ-12)[38]<br><br>Wellbeing: Warwick Edinburgh Mental Wellbeing Scale (WEMWBS)[39]<br><br>Hormone function: Levels of cortisol in response to awakening and throughout the day<br><br>Immune function: C-reactive protein<br><br>2.<br>EuroQoL (EQ-5D-5L)[29]<br>Trimbos/iMTA Questionnaire for Costs Associated with Psychiatric Illness (TiC-P)[30]<br>Client Service Receipt Inventory[31]

Tertiary	1. Do psychiatric, personality, trauma and social support factors at baseline (social support, trauma exposure, anxiety, age, gender, education, neuroticism, past and current psychiatric status, immune and hormone function) influence (i.e., moderate) levels of symptoms (PTSD or MD) psychological distress and wellbeing at follow-up?<br><br>2. Do changes in resilience-related factors (rumination, responses to intrusions, concrete thinking, resilience appraisals, practice of iCT-R/Mind-Online tools) mediate symptom levels of PTSD and MD at one year follow-up with iCT-R and Mind-Online?<br><br>3. Do changes in resilience-related factors (rumination, responses to intrusions, concrete thinking, resilience appraisals, practice of iCT-R/Mind-Online tools) mediate symptom levels of PTSD and MD at two year follow-up with iCT-R and Mind-Online?	1.<br>Neuroticism: Eysenck Personality Questionnaire, Neuroticism subscale (EPQ)[35]<br>Social Support (SS)[adapted from 36]<br>Age, gender, education: Demographic Questionnaire (unpublished)<br>Psychological Distress: GHQ[38]<br>Wellbeing: WEMWBS[39]<br>Immune function: CRP<br>Hormone function: Cortisol<br><br>1., 2., and 3<br>Past & Current Psychiatric Status: Structured Clinical Interview for DSM-V[24]<br>Symptoms PTSD: PCL-5[22]<br>Symptoms Depression: PHQ-9[23]<br>Anxiety: GAD-7[26]<br>Trauma Exposure: Trauma Screener (unpublished)<br><br>2. and 3.<br>Rumination: Ruminative Response Scale[37] (Brooding Subscale)<br>Intrusions: Response to Intrusions Questionnaire (RIQ)[33,34], short version<br>Duration, Frequency, Distress Linked to Intrusions (unpublished)<br>Concrete Thinking: Concrete & Abstract Thinking (unpublished)<br>Practice iCT-R/Mind Online tools (unpublished)<br>Resilience: CD-RISC[25] & Resilience Scale (Wagnild & Young)[40]

	4. Does concrete thinking, practice of tools and responses to intrusions at 6 months predict diagnoses and levels of PTSD and depression symptoms at one year?	4.<br>Intrusions: Response to Intrusions Questionnaire (RIQ)[33,34], short version<br>Duration, Frequency, Distress Linked to Intrusions (unpublished)<br>Concrete Thinking: Concrete & Abstract Thinking (unpublished)<br>Practice iCT-R/Mind Online tools (unpublished)
Treatment conditions	1. Supported Internet-delivered cognitive training for resilience (iCT-R)<br>2. Supported Internet-delivered mental health education (Mind-Online)<br>3. 24 months standard practice	

Abstract

Introduction: Emergency workers dedicate their lives to promoting public health and safety yet suffer higher rates of post-traumatic stress disorder (PTSD) and major depression (MD) compared to the general population. They also suffer an associated increased risk for physical health problems, which may be linked to specific immunological and endocrine markers or changes in relevant markers. Poor physical and mental health is costly to organisations, the NHS and society. Existing interventions aimed at reducing risk of mental ill health in this population are not very successful. More effective preventative interventions are urgently needed. Through our systematic research approach, we first conducted a large-scale prospective study of newly recruited student paramedics, identifying two cognitive factors (rumination and resilience appraisals) that predicted episodes of PTSD and MD over a two-year period. We then developed an innovative intervention to modify cognitive predictors. This protocol is for a randomized controlled trial to evaluate the efficacy of the resilience intervention. We compare the new resilience intervention to an existing programme and to standard practice.

Design, methods and analysis: A total of N=570 student paramedics will be recruited from five participating universities. They will be randomly allocated to one of the two online training programmes or to standard practice. Follow-up will occur after the training/standard practice period, and at one and two years. We hypothesise that the new intervention will lead to reduced rates of subsyndromal and full syndromal PTSD and MD, cost savings to the NHS and society, and reduced levels of markers of inflammation and the stress hormone cortisol.

Ethics and dissemination: The Medical Sciences Inter-Divisional Research Ethics Committee granted ethical approval on 17 August, 2017 at the University of Oxford, valid until 31 December, 2020. Reference: R44116/RE001.

BACKGROUND AND RATIONALE

Emergency workers carry a threefold increase, compared to the general population, in risk for major depression (MD) and posttraumatic stress disorder (PTSD), and an associated increased risk of poor physical health (1). To date, interventions aimed at reducing risk of ill mental health in this population have been unsuccessful. Randomized controlled trials (RCT) found that trauma risk management, a peer support system widely available to the police and ambulance services in England (2); critical incident stress debriefing widely used by UK fire-services (3), and the charity Mind’s six-session group-based resilience intervention had no effect on resilience or rates of mental ill health (4) (Table 2). More effective preventative interventions for emergency workers are urgently needed.

Established interventions have likely been unsuccessful because they fail to target predictors of mental ill health and are offered to emergency workers after rather than before repeated exposure to the stresses linked to their work. Moreover, cognitive strategies that could help them cope with characteristic stressors are not included as part of the training. Our and others’ research has demonstrated that exposure to trauma or stressful scenarios through imagery reduces anxiety for police officers (9) and other at risk populations (10). Development of more effective interventions requires identification of predictors of mental disorders and an understanding of how to modify them.

In a series of experimental and prospective studies, we identified two cognitive factors that are particularly powerful predictors of poor mental health in emergency workers: rumination (repetitive negative thinking) and resilience appraisals. Those who reported ruminative thoughts during critical incidents were more likely to experience poor levels of coping (5). Adaptive appraisals during analogue trauma lead to more successful attempts to regulate emotions and fewer PTSD symptoms (6). Our large-scale prospective study of newly recruited paramedics (1) investigated predictors of PTSD and depression derived from cognitive theories of PTSD and depression. Rumination at the start of paramedic training uniquely predicted PTSD; low resilience uniquely predicted an episode of major depression.

We then developed an intervention to modify peri-traumatic ruminative thinking (i.e, thinking repetitively in an abstract way during trauma). Training to think in a concrete style (e.g., focusing on objective details and the sequence of events) led to significantly fewer intrusive memories and PTSD symptoms than individuals trained in a ruminative style (7). We also applied one of the core techniques of our successful treatment for PTSD, updating the memory of the stressful event with helpful information, as a preventative strategy in analogue trauma and found that it is more helpful in reducing repetitive thinking and PTSD symptoms than control interventions including exposure (8).

Research has further demonstrated that exposure to trauma or stressful scenarios through imagery reduces anxiety for police officers (9) and other at risk populations (10) and that internet-based cognitive treatment that includes attention training as a core component significantly reduces anxiety (11).

Neurobiological Factors linked to PTSD and MD

Genetic (12) and longitudinal (13) studies suggest that inflammation is a pre-existing vulnerability factor for the development of PTSD in trauma-exposed individuals rather than simply a correlate of subjective distress, disease severity, or maladaptive coping strategies following PTSD onset. For example, brain imaging studies have shown that high inflammation levels may increase threat perception (negative valence). Peripheral administration of lipopolysaccharides (LPS), residues from bacterial cells' components known to elicit a strong systemic inflammatory response, potentiates amygdala activity in response to socially threatening stimuli (fear faces) (14). In turn, greater pre-treatment amygdala reactivity to threat predicts less symptom reduction during CBT (15). Additionally, inflammation is an important risk factor for depression (16) and cardiovascular disease (17), which frequently accompany PTSD (18). Our study will investigate the link between inflammation and the development of PTSD and MD in trauma-exposed student paramedics. We will investigate whether or not our active intervention can reduce levels of clinically-relevant inflammation levels, such as C-Reactive Protein (CRP), known to increase risk of psychiatric as well as cardiovascular and metabolic conditions comorbid with PTSD and MD.

Given the wealth of literature supporting a relationship between the stress hormone, cortisol, and PTSD and MD, we will also systematically assess the cortisol awakening response (CAR) and diurnal cycle. The CAR is an endocrine marker, defined as the change in cortisol concentration that occurs during the first hour after waking from sleep (19). A meta-analysis (19) of 62 studies concluded that increases in the CAR were associated with job stress and life stress and linked to greater fatigue, burnout and exhaustion and risk for later health states, such as coronary heart disease. A recent study (20) found that higher CAR predicted future episodes of major depression within a 2.5 year period. We anticipate that the intervention will reduce the CAR and cortisol throughout the day and protect against the development of PTSD and MD.

OBJECTIVES AND OUTCOME MEASURES

Objectives	Outcome Measures	Timepoint(s) of evaluation of this outcome measure (if applicable)
**Primary Objectives**<br><br>**1**. Is internet-delivered cognitive training for resilience (iCT-R) more effective than accessing already available mental health information (Mind-Online) or having no specific training (standard practice), i.e., does iCT-R lead to fewer cases of PTSD and Major Depression (including subsyndromal PTSD and MD) and less PTSD and MD symptomatology at follow-up compared to Mind-Online and standard practice?	1.<br>Depression: PHQ-9[23]<br>PTSD: PCL-5[22]<br>Structured Clinical Interview for DSM-5 – PTSD and MD modules [24]	1.<br>Baseline<br>6 weeks<br>52 weeks<br>104 weeks
**Secondary Objectives**<br><br>**1.** Does internet-delivered cognitive training for resilience (iCT-R) lead to greater improvement in secondary outcome measures (resilience, rumination, hormone and immune function, smoking, weight gain, alcohol use, symptoms of anxiety, and sleep problems, psychological distress, wellbeing) than Mind-Online and standard practice?<br><br>**2.** Is iCT-R more cost-effective than Mind-Online or standard practice, i.e., is the cost per	1.<br>Resilience: CD-RISC[25], Wagnild & Young[40]<br>Rumination: RRS[37] (Brooding) & RIQ[33]<br>Anxiety: GAD-7[26]<br>Smoking & Alcohol Use: Smoking & Alcohol Use (unpublished)<br>Weight: Weight Questionnaire (unpublished)<br>Sleep problems: ISI[27]<br>Psychological distress: General Health Questionnaire (GHQ-12)[38]<br>Wellbeing: WEMWBS[39]<br>Hormone function: cortisol levels<br>Immune function: CRP levels<br><br>**2.**<br>Quality of life: EQ-5D-5L[29]<br>Costs: Trimbos/iMTA-TiC-P[30], Client	1.<br>Baseline<br>6 weeks<br>52 weeks<br>104 weeks

participant without an episode or symptoms of PTSD or MD and costs per QALY gained smaller?	Service Receipt Inventory[32]	2. Baseline<br>6 weeks<br>52 weeks<br>104 weeks
**Tertiary Objectives**<br><br>1. Do psychiatric, personality, trauma and social support factors at baseline (social support, trauma exposure, anxiety, age, gender, education, neuroticism, past and current psychiatric status, immune function) influence (i.e., moderate) levels of symptoms (PTSD or MD) psychological distress and wellbeing at follow-up?<br><br>2. Do changes in resilience-related factors (rumination, responses to intrusions, concrete thinking, resilience appraisals, practice of iCT-R/Mind-Online tools) mediate symptom levels of PTSD and MD at one year follow-up with iCT-R and Mind-Online?<br><br>3. Do changes in resilience-related factors (rumination, responses to intrusions, concrete thinking, resilience appraisals, practice of iCT-R/Mind-Online tools) mediate symptom levels of PTSD and MD at two year follow-up with iCT-R and Mind-Online?<br><br>4. Does concrete thinking, practice of tools and responses to intrusions at 6 months predict diagnoses and levels of PTSD and depression symptoms at one year?	1.<br>Neuroticism: Eysenck Personality Questionnaire, Neuroticism subscale (EPQ)[35]<br>Social Support (SS)[adapted from 36]<br>Age, gender, education: Demographic Questionnaire (unpublished)<br>Psychological Distress: GHQ[38]<br>Wellbeing: WEMWBS[39]<br>Immune function: CRP<br>Hormone function: Cortisol<br><br>1., 2., 3 and 4<br>Past & Current Psychiatric Status: Structured Clinical Interview for DSM-V[24]<br>Symptoms PTSD: PCL-5[22]<br>Symptoms Depression: PHQ-9[23]<br>Anxiety: GAD-7[26]<br>Trauma Exposure: Trauma Screener (unpublished)<br><br>2. and 3.<br>Rumination: Ruminative Response Scale[37] (Brooding Subscale)<br>Intrusions: Response to Intrusions Questionnaire (RIQ)[33,34], short version<br>Duration, Frequency, Distress Linked to Intrusions (unpublished)<br>Concrete Thinking: Concrete & Abstract Thinking (unpublished)<br>Practice iCT-R/Mind Online tools (unpublished)<br>Resilience: CD-RISC[25] & Resilience	1.<br>Baseline<br>6 weeks<br>52 weeks<br>104 weeks<br><br>2. and 3.<br>Baseline<br>6 weeks<br>52 weeks<br>104 weeks<br><br>4.<br>Baseline<br>6 weeks<br>24 weeks

	Scale (Wagnild & Young)[40]<br>4.<br>Intrusions: Response to Intrusions Questionnaire (RIQ)[33,34], short version<br>Duration, Frequency, Distress Linked to Intrusions (unpublished)<br>Concrete Thinking: Concrete & Abstract Thinking (unpublished)<br>Practice iCT-R/Mind Online tools (unpublished)	

6. TRIAL DESIGN

The design is a single blind (assessors blinded) randomised controlled trial comparing a supported internet-delivered resilience training to alternative online training (Mind-Online) and standard practice control, with an embedded process study.

7. PARTICIPANT IDENTIFICATION

7.1. Trial Participants

Participants will be adults training to be student paramedics at the University of Brighton, Oxford-Brookes University, University of Bournemouth, University of Worcester and University of Hertfordshire.

7.1.1 Inclusion Criteria

- Aged 18 and above.
- In years 1, 2 or 3 of student paramedic training.
- Access to internet.
- Willing to be randomly allocated.

7.1.2. Exclusion Criteria

- Current symptoms of PTSD or MD requiring treatment

8. TRIAL PROCEDURES

8.1 Recruitment

Participants will be recruited at five sites:

1. University of Worcester
2. University of Brighton
3. Oxford-Brookes University
4. Bournemouth University
5. University of Hertfordshire

The research assistant will present the study to the Year 1, 2, and 3 cohorts at collaborating universities. Interested students will be asked to provide their email address so that a link may be emailed to them which includes the Participant Information Sheet (PIS). The PIS contains the contact details of the researchers so potential recruits may contact them with any queries. Interested participants will be invited to ask any questions.

If participants decide that they would like to take part, they will have the option of giving consent, online, or the option to ask further questions. If they endorse the latter, the researcher will email the participant to schedule a time to speak on the telephone to answer their questions.

Once participants' questions have been answered and if they wish to give consent or if they have no questions and wish to give their consent, they will be required to confirm that they understand the various points of the study and electronically signing their name. Once they have given consent online, they will be asked to complete the two screening questionnaires. It will be made clear that participation is entirely voluntary and that volunteers may withdraw from the study at any point without incurring any negative consequences.

8.2 Screening and Eligibility Assessment

Once participants have given consent, they will complete two screening questionnaires (PCL-5 and PHQ-9) to assess eligibility. Participants who score in the clinical range on measures of post-traumatic stress and depression will be contacted by the trained research assistant. If their symptoms are interfering with their life and they would like treatment, the research assistant will offer them information on how to access evidence-based treatment for these conditions in local services. They will be excluded from the trial.

Eligible participants who wish to participate and have given consent will be emailed a link to complete their initial baseline questionnaires and an appointment will be made to collect a plasma sample and give them their saliva collection tubes.

8.3 Informed Consent

After receiving detailed written and verbal information about the exact nature of the trial and what it will involve for them, and if agreeing to participate in the trial, the participant will electronically sign their name on the latest approved version of the consent form. They will complete the two screening questionnaires (PCL-5 and PHQ-9) and if eligible, they will personally sign the consent form when they meet the research assistant at their face-to-face appointment for the plasma sample, which will be scheduled within one week of signing the electronic consent form.

The information sheet and consent form state the participant is free to withdraw from the trial at any time for any reason without penalty, by advising the researchers of this decision.

The participant will be allowed as much time as wished to consider the information, and the opportunity to question the Investigator or other independent parties to decide whether they will participate in the trial. Electronic informed consent will be obtained prior to completing the two screening questionnaires. Written informed consent will be obtained at the first meeting with the participant, which will be scheduled within one week of completing the screening questionnaires. Written informed consent will include participant dated signature and dated signature of the person who presented and obtained the informed consent. The person who obtained the consent must be suitably qualified and

experienced, and have been authorised to do so by the Chief/Principal Investigator. The original signed form will be retained at the trial site.

8.4 Randomisation

Random allocation to the three trial conditions (iCT, Mind-Online, standard practice) will be at a 1:1:1 ratio, stratified by site and initial eligibility (above or below cut-off on PHQ-9 and PCL-5 at screening) by the Oxford Clinical Trials Research Unit (OCTRU).

8.5 Subsequent Assessments

Participants will complete assessments with an independent trained assessor who will be unaware of trial condition at baseline, 6, 52 and 104 weeks. They will also complete questionnaires on these occasions.

8.6 Discontinuation/Withdrawal of Participants from Trial Treatment

Each participant has the right to withdraw from the trial at any time. The reason for withdrawal will be recorded.

8.7 Definition of End of Trial

The end of trial is the date of the last follow up with the last participant.

9. INTERVENTIONS

Internet-delivered cognitive training for resilience (iCT-R) and Mind-Online will be delivered over 6 weeks, with the support of the trained research assistant (wellbeing coach) by email, SMS and phone calls (on average taking up to 1 hour of research assistant time for both treatments). Participants will be able to print the modules they complete. The research assistant will send top up reminders every month and every 6 months, participants will have the opportunity to talk with the research assistant about putting their tools in place or accessing and practising the wellbeing information they have learned.

9.1 Internet-delivered cognitive training for resilience (iCT-R)

iCT-R follows a similar format to the internet-based programmes we have developed for social anxiety[11] and PTSD[21]. The core information is delivered in modules of which there are six. The modules include whiteboard videos to explain concepts, audio files for practicing concrete thinking, testimony from qualified paramedics and video footage of student paramedic call-outs for use in the modules' experiential exercises. The modules are as follows:

- It Matters What You Focus On: Helpful and Unhelpful Attention
- Get Out of Your Head: Helpful and Unhelpful Thinking
- Habits and Dwelling: How to Change Them
- Dealing with Unwanted Memories: Then vs Now
- Dealing with Worry
- My Blueprint

9.2 Mind-Online

The alternative resilience programme is Mind Online, a series of six modules available online covering information and advice about wellbeing and mental health topics, which have demonstrated high acceptability to emergency workers in a previous trial (4). The six modules are:

- Dealing with Stress
- Sleep Problems
- Anger
- Depression
- Post-traumatic Stress Disorder
- Mindfulness

Participants will receive the same frequency, type and duration of remote support as in iCT-R.

9.3 Treatment fidelity

Treatment fidelity will be assessed by an independent rater. The content of a random sample of email communications will be scored for reference to content relevant to each training programme.

9.4 Standard Practice

Participants initially allocated to the standard practice will we be offered iCT-R at 104 weeks.

10. SAFETY REPORTING

10.1 Definitions

**Term**	**Definition**
**Adverse Event (AE)**	Any untoward medical occurrence in a participant or clinical trial participant and which are not necessarily caused by or related to that product
**Serious Adverse Event (SAE)**	Any adverse event that -<br>• Results in death<br>• Is life-threatening*<br>• Required hospitalisation or prolongation of existing hospitalisation**<br>• Results in persistent or significant disability or incapacity<br>• Consists of a congenital anomaly or birth defect<br>• Other medically important condition***

***Note:** The term 'life-threatening' in the definition of serious refers to an event in which the trial participant was at risk of death at the time of the event or it is suspected that used or continued used of the product would result in the subject's death; it does not refer to an event which hypothetically might have caused death if it were more severe.

10.2 Causality

The Principal Investigator will assess each SAE to determine the causal relationship:

**Relationship**	**Description**	**Reasonable possibility that the SAE may have been caused by the intervention?**
**Unrelated**	There is no evidence of any causal relationship with the trial/intervention	No
**Unlikely**	There is little evidence to suggest there is a causal relationship with the trial/intervention (e.g. the event did not occur within a reasonable time after the intervention). There is another reasonable explanation for the event (e.g. the participant's clinical condition, other concomitant treatment).	No
**Possible**	There is some evidence to suggest a causal relationship with the trial/intervention (e.g. because the event occurs within a reasonable time after the intervention). However, the influence of other factors may have contributed to the event (e.g. participant's physical health or exposure to critical incident at work).	Yes
**Probable**	There is evidence to suggest a causal relationship and the influence of other factors is unlikely.	Yes
**Definite**	There is clear evidence to suggest a causal relationship and other possible contributing factors can be ruled out.	Yes

10.3. Expectedness

The Chief Investigator will assess each SAE to perform the assessment of expectedness. The expectedness assessment should be made according to the information as detailed in the protocol.

<u>Reference Safety Information (RSI)</u>

There are no expected side effects linked to either intervention.

Expectedness decisions must not take into account factors such as the participant population and participant history. Expectedness is not related to what is an anticipated event within a particular disease. SAEs which add significant information on specificity or severity of a known, already documented adverse event constitute unexpected events.

10.4 Procedures for Recording Adverse Events

All AEs occurring during the trial that are observed by the research team or reported by the participant, will be recorded. The following information will be recorded: description, date of onset and end date, severity, assessment of relatedness to intervention, and action taken. Follow-up information should be provided as necessary. The severity of events will be assessed on the following scale: 1 = mild, 2 = moderate, 3 = severe.

10.5 Reporting Procedures for Serious Adverse Events

All SAEs must be reported on the SAE reporting form to the REC within 15 working days of the Principal Investigator becoming aware of the event, using the Health Research Authority safety report form for a non-Clinical Trial of an Investigation of a Medicinal Product (non-CTIMP).
It will also be reviewed at the next Trial Steering Committee meeting. All SAE information must be recorded on an SAE form and faxed, or scanned and emailed, to the REC. Additional and further requested information (follow-up or corrections to the original case) will be detailed on a new SAE Report Form and faxed/emailed to the REC.

10.6 Monitoring for negative effects of treatment delivery over the internet

We will assess participant's evaluation of the internet modules and design through a feedback and rating form at the end of each module and in comments to the research assistant in emails or telephone calls.

11. STATISTICS

11.1. Description of Statistical Methods

11.1.1. Main Analysis

All analyses will be intent-to-treat. No interim analyses are planned. We will compare rates of PTSD and MD in each training condition using Chi square analysis. Continuous measures will be analysed using hierarchical linear modeling. This analysis models random slopes and intercepts for participants, and tests the fixed effects of repeated assessments over time (level 1) and training condition (level 2) using data from all participants. It takes into account that participants are nested within site (level 3). Variables will be centred for the analysis.

11.1.2. Biological Analyses

The relationship between symptom levels (PTSD and MD) and levels of salivary cortisol and C-reactive protein at post-intervention and follow-up will be analysed with hierarchical linear modelling.

11.1.2 Process Evaluation

A process evaluation conducted alongside the main trial will explore moderators of outcome and mediators of change. Example moderators include social support, number of traumatic events, anxiety, age, gender, years of education, neuroticism, baseline CRP and CAR, past psychiatric history, ratings of treatment helpfulness, and fidelity. Mediators of training

outcome to be considered are processes hypothesized to predict PTSD and MD in paramedics (resilience appraisals, rumination and responses to unwanted memories).

11.1.3 Health Economic Evaluation

A full economic evaluation will be performed to compare the costs and effects of providing iCT-R versus the placebo (already available health information). These will be performed by Dr Apostolos Tsiachristas. Data collected and the existing literature will be used in a decision analytic model to calculate the incremental costs per quality adjusted life years (QALYs) during the participant's lifetime. Uncertainty in the results will be addressed in sensitivity analyses and displayed in cost-effectiveness planes and cost-effectiveness acceptability curves.

11.2 The Number of Participants

We plan to recruit 570 participants.

The risk of student paramedics developing full syndromal PTSD and MD over two years without intervention is 10% (1), and 25% if subsyndromal PTSD and MD that cause significant distress are included. We predict that our intervention will reduce the relative risk by 50%. Setting power at 80%, α=.05 and hypothesizing a reduction of relative risk of 50% gives an odds ratio of 0.429, which requires a total sample size of N=304 to show a risk reduction of 50% between the active and placebo intervention. Thus, each condition would require N=152. Since we have a third condition (no-training control), the total sample size required would be N=456. Allowing for a 20% rate of attrition, we will require a total sample size of N=570.

11.3 The Level of Statistical Significance

Significance levels were set at $p < .05$.

11.4 Criteria for the Termination of the Trial

There are no stopping rules for the trial as it is a low risk non-CTIMP.

11.5 Inclusion in Analysis

Analyses will be intent-to-treat, i.e. all randomized participants will be included in the analysis.

12. DATA MANAGEMENT

12.1. Source Data

Source data will be captured online via Qualtrics software. Access to the system will be restricted to named study personnel only and via password protection. We have arranged with the University of Oxford IT services for the files to be encrypted and backed up on a weekly basis using the Tivoli Storage Manager, the data are copied to three separate tapes. One copy resides in the Tape Robot in the IT Services Machine room. The other two copies are held in locked fireproof safes, one onsite at IT Services, one offsite in locked premises. The data on the tapes are inaccessible without the TSM database. The data on the offsite tapes are encrypted.

12.2. Access to Data

Direct access will be granted to authorised representatives from the host institution and the regulatory authorities to permit trial-related monitoring, audits and inspections.

13. QUALITY ASSURANCE PROCEDURES

The trial will be conducted in accordance with the current approved protocol, relevant regulations and standard operating procedures. Regular monitoring will be performed. Data will be evaluated for compliance with the protocol, and completeness and accuracy in relation to source documents (e.g., for SCID-5). The trial steering committee will regularly view recruitment rates, compliance with delivery of different training programmes and completeness of data collection.

14. ETHICAL AND REGULATORY CONSIDERATIONS

14.1. Declaration of Helsinki

The Investigator will ensure that this trial is conducted in accordance with the principles of the Declaration of Helsinki.

14.2. Guidelines for Good Clinical Practice

The Investigator will ensure that this trial is conducted in accordance with relevant regulations and with Good Clinical Practice.

14.3. Approvals

The protocol, informed consent form, participant information sheet and advertising material has been submitted and approved by the University of Oxford Inter-Divisional Medical Sciences Research Ethics Committee (REC).

14.4. Participant Confidentiality

The trial staff will ensure that the participants' anonymity is maintained. The participants will be identified only by a participant ID number on all trial documents and any electronic database, with the exception of the signed consent form, where participant initials may be added. All documents will be stored securely and only accessible by trial staff and authorised personnel. The trial will comply with the Data Protection Act, which requires data to be anonymised as soon as it is practical to do so.

14.6. Expenses and Benefits

Participants will be paid 30 pounds for their time at 104 weeks.

15. FINANCE AND INSURANCE

15.1. Funding

The trial is funded by MQ, awarded to Jennifer Wild and Anke Ehlers.

15.2. Insurance

The University has a specialist insurance policy in place which would operate in the event of any participant suffering harm as a result of their involvement in the research (Newline Underwriting Management Ltd, at Lloyd's of London).

16. PUBLICATION POLICY

The results of the trial will be published in peer-reviewed international journals and will be made open access.

17. REFERENCES

1. Wild, J., Smith, K.V., Thompson, E., Bear, F., Lommen, M. & Ehlers, A. (2016). A prospective study of pre-trauma risk factors or posttraumatic stress disorder and depression. *Psychological Medicine*.

2. Greenberg, N., Langston, V., Everitt, B., Iversen, A., Fear, N.T., Jones, N. & Wessely, S. (2010). A cluster randomized controlled trial to determine the efficacy of trauma risk management (TRiM) in a military population. *Journal of Traumatic Stress, 23,* 430-36.

3. van Emmerik, A.A., Kamphuis, J.H., Hulsbosch, A.M., & Emmelkamp, P.M. (2002). Single session debriefing after psychological trauma: a meta analysis. *Lancet, 7,* 360, 766-71.

4. Wild, J., El-Salahi, S., Degli Esposti, M. & Ehlers, A. (In preparation). A randomised controlled trial to evaluate Mind's resilience intervention for emergency service workers.

5. Shepherd, L. & Wild, J. (2013). Cognitive appraisals, objectivity, and coping in ambulance workers: a pilot study. *Emergency Medicine Journal, 31,* 41-4.

6. Shepherd, L. & Wild, J. (2014). Emotion regulation, physiological arousal and PTSD symptoms in trauma-exposed individuals. *Journal of Behavior Therapy and Experimental Psychiatry, 45,* 360-7.

7. White, R. & Wild, J. (2015). "Why" or "How": The Effect of Concrete Versus Abstract Processing on Intrusive Memories Following Analogue Trauma. *Behavior Therapy.*

8. Pile, V., Barnhofer, T., & Wild, J. (2015). Updating versus exposure to prevent consolidation of conditioned fear. *PLoS ONE, 10,* 1-21.

9. Arnetz, B.B., Arble, E., Backman, L., Lynch, A., & Lublin, A. (2013). Assessment of a prevention program for work-related stress among urban police officers. *Internatonal Archives of Occupational and Environmental Health, 86*, 79-88.

10. Wild, J., Hackmann, A., & Clark, D.M. (2008). Rescripting early memories linked to negative images in social phobia: A pilot study. *Behavior Therapy, 39,* 47-56.

11. Stott, R., Wild, J., Grey, N., Liness, S., Warnock-Parkes, E., Commins, S., Reading, J., Bremner, G., Woodward, E., Ehlers, A. & Clark, D.M. (2014). Internet-delivered cognitive therapy for social anxiety disorder: a development series. *Behavioural and Cognitive Psychotherapies, 41*, 383-97.

12. Michopoulos, V., Rothbaum, AO., Jovanovic, T., Almli, LM., Bradley, B., Rothbaum, BO., Gillespie, CF., & Ressler, KJ. (2015). Association of CRP genetic variation and CRP level with elevated PTSD symptoms and physiological responses in a civilian population with high levels of trauma. *American Journal of Psychiatry,* 172, 353-362.

13. Eraly, SA., Nievergelt, CM., Maihofer, AX., Barkauskas, DA., Biswas, N., Agorastos, A., O'Connor, DT., Baker, DG., Marine Resiliency Study Team (2014). Assessment of plasma C-reactive protein as a biomarker of posttraumatic stress disorder risk. *JAMA Psychiatry*, 71, 423-431.

14. Inagaki, TK., Keely, A., Muscatell, MA., Irwin, MR., Cole, SW., & Eisenberger, NI. (2012). Inflammation Selectively Enhances Amygdala Activity to Socially Threatening Images. *Neuroimage, 15*, 3222-3226.

15. Bryant, RA., Felmingham, K., Kemp, A., Das, P., Hughes, G., Peduto, A., & Williams, L. (2008). Amygdala and ventral anterior cingulate activation predicts treatment response to cognitive behaviour therapy for post-traumatic stress disorder.*Psychological Medicine, 38,* 555-561.

16. Miller, AH. & Raison, CL. (2016). The role of inflammation in depression: from evolutionary imperative to modern treatment target. *Nature Reviews in Immunology*, 16, 22-34.

17. Ridker, PM., Cushman, M., Stampfer, MJ., Russell, PT., & Hennekens, CH. (1997). Inflammation, Aspirin, and the Risk of Cardiovascular Disease in Apparently Healthy Men. *New England Journal of Medicine, 336*, 973-979.

18. Sumner, JA., Kubzansky, LD., Elkind, MS., Roberts, AL., Agnew-Blais, J., Chen, Q., Cerda, M., Rexrode, KM., Rich-Edwards, JW., Spiegelman, D., Suglia, SF., Rimm, EB., & Koenen, KC. (2015). Trauma Exposure and Posttraumatic Stress Disorder Symptoms Predict Onset of Cardiovascular Events in Women. *Circulation*, 28, 251-259.

19. Chida, Y. & Steptoe, A. (2009). Cortisol awakening response and psychosocial factors: a systematic review and meta-analysis. Biological Psychiatry, 80, 265-278.

20. Vrshek-Schallhorn, S., Doane, LD., Mineka, S., Zinbarg, RE., Craske, MG., & Adam, EK. (2013). The cortisol awakening response predicts major depression: predictive stability over a 4-year follow-up and effect of depression history. *Psychological Medicine, 43,* 483-493.

21. Wild, J., Warnock-Parkes, E., Grey, N., Stott, R., Wiedemann, M., Canvin, L., ... & Ehlers, A. (2016). Internet-delivered cognitive therapy for PTSD: A development pilot series. *European journal of psychotraumatology*, *7*(1), 31019.

22. Weathers FW, Litz BT, Keane TM, Palmieri PA, Marx BP, Schnurr PP. The PTSD Checklist for DSM-5 (PCL-5).

23. Kroenke K, Spitzer RL, Williams JBW. The PHQ-9. *Journal of General Internal Medicine.* 2001;16(9):606-613.

24. First MB, Williams, JBW, Karg RS, Spitzer RL (2017). Structured Clinical Interview for DSM-5® Disorders—Clinician Version (SCID-5-CV). American Psychiatric Association Publishing.

25. Connor, K.M. & Davidson, J.R.T. (2003). Development of a new resilience scale: the Connor-Davidson resilience scale (CD-RISC). *Depression and Anxiety,* ***18,*** 76-82.

26. Spitzer Rl, Kroenke K, Williams JW, Löwe B. A brief measure for assessing generalized anxiety disorder: The GAD-7. *Archives of Internal Medicine.* 2006;166(10):1092-1097

27. Morin CM, Belleville G, Bélanger L, Ivers H. The Insomnia Severity Index: psychometric indicators to detect insomnia cases and evaluate treatment response. Sleep. 2011;34(5):601-8.

28. Babor, TF., Higgins-Biddle, JC., Saunders, JB, Monteiro, MG. (2001). The Alcohol Use Disorders Identification Test, Guidelines for Use in Primary Care, Second Edition, Department of Mental Health and Substance Dependence, World Health Organization.

29. Whynes DK, Group T. Responsiveness of the EQ-5D to HADS-identified anxiety and depression. *J Eval Clin Pract.* 2009;15(5):820-825.

30. Bouwmans C, Krol M, Severens H, Koopmanschap M, Brouwer W, Roijen LH. The iMTA Productivity Cost Questionnaire: A Standardized Instrument for Measuring and Valuing Health-Related Productivity Losses. *Value Health.* 2015;18(6):753-758.

31. Rapaport MH, Clary C, Fayyad R, Endicott J. Quality-of-life impairment in depressive and anxiety disorders. *Am J Psychiatry.* 2005;162(6):1171-1178.

32. Chisholm D, Knapp MR, Knudsen HC, Amaddeo F, Gaite L, van Wijngaarden B. Client Socio-Demographic and Service Receipt Inventory--European Version: development of an instrument for international research. EPSILON Study 5. European Psychiatric Services: Inputs Linked to Outcome Domains and Needs. *Br J Psychiatry Suppl.* 2000(39):s28-33.

33. Clohessy, S. & Ehlers, A. (1999). PTSD symptoms, response to intrusive memories, and coping in ambulance service workers. *British Journal of Clinical Psychology, 38*, 251-265.

34. Murray, J., Ehlers, A. & Mayou, R.A. (2002). Dissociation and posttraumatic stress disorder: Two prospective studies of road traffic accident victims. *British Journal of Psychiatry, 180*, 363-368.

35. Eysenck, H. J. & Eysenck, S. B. G. (1975). *Manual of the Eysenck Personality Questionnaire.* London: Hodder & Stoughton.

36. Sarason, I.G., Sarason, B.R.., Shearin, E.N., & Pierce, G.R. (1987). A brief measure of social support: Practical and Theoretical implications. *Journal of Social and Personal Relationships, 4,* 497-510.

37. Treynor, W., Gonzalez, R. & Nolen-Hoeksema, S. (2003). Rumination reconsidered: a psychometric analysis. *Cognitive Therapy and Research, 27,* 247-259.

38. Golderberg D, Williams P (1988). A user's guide to the General Health questionnaire. Windsor, UK: NFER-Nelson.

39. Tennant R, Hiller L, Fishwick R, Platt P, Joseph S, Weich S, Parkinson J, Secker J, Stewart-Brown S (2007) The Warwick-Edinburgh Mental Well-being Scale (WEMWBS): development and UK validation, Health and Quality of Life Outcome; 5:63 doi:101186/1477-7252-5-63

40. Wagnild, G. M., & Young, H. M. (1993). Development and psychometric evaluation of the Resilience Scale. *Journal of nursing measurement*.

15. Trial Flow Chart

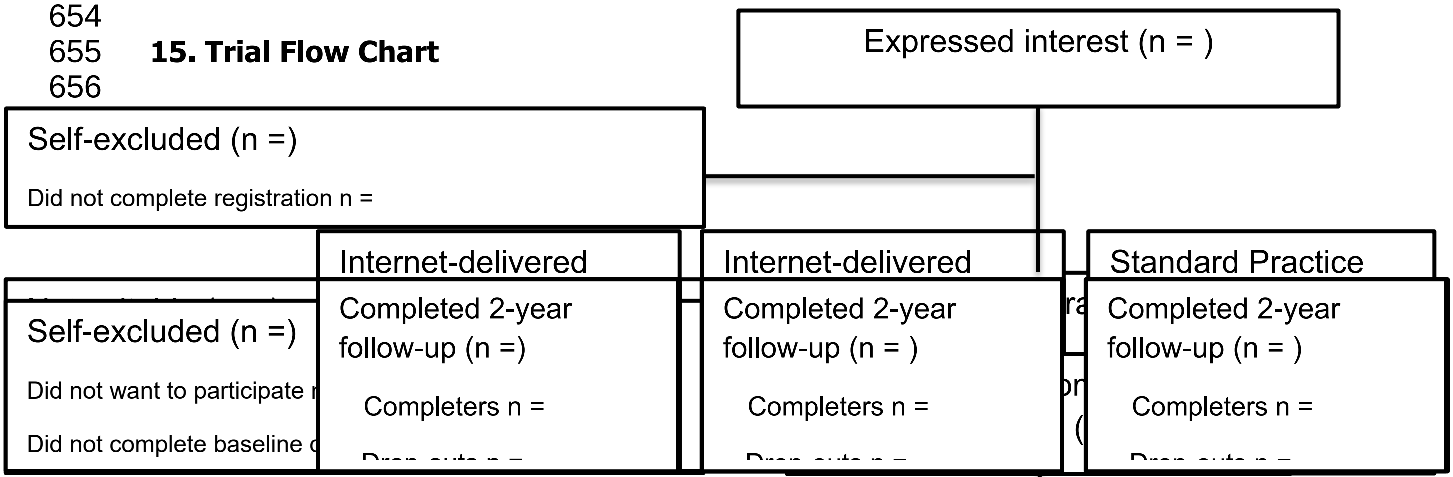

APPENDIX B: SCHEDULE OF PROCEDURES

**Procedures**	**Assessments**				
	**Baseline**	**6-weeks**	**24-weeks**	**52-weeks**	**104-weeks**
**Informed consent**	X				
**Screening**					
PHQ-9	X	X	X	X	X
PCL-5	X	X	X	X	X
**Randomisation**	X				
**Interviews**					
Structured Clinical Interview DSM-5: PTSD & MD modules	X	X		X	X
**Questionnaires**					
CD-RISC	X	X		X	X
Wagnild	X	X		X	X
GAD-7	X	X		X	X
Smoking & Alcohol	X	X		X	X
Weight	X	X		X	X
ISI	X	X		X	X
GHQ	X	X		X	X
WEMWBS	X	X		X	X
EQ5D5L	X	X		X	X
Trimbos/iMTA-TiC-P	X	X		X	X
CSRI	X	X		X	X
EPQ	X			X	X
SS	X	X		X	X
RRS (Brooding)	X	X		X	X
RIQ	X	X		X	X
TS	X	X	X	X	X
Duration, Frequency, Distress linked to intrusions	X	X	X	X	X
Concrete & Abstract Thinking	X	X	X		
**Biological samples**					
Cortisol	X	X		X	X
CRP	X	X		X	X
**Compliance**					
Practice iCT-R/Mind-Online tools		X	X	X	X

6. APPENDIX C: AMENDMENT HISTORY

**Amendment No.**	**Protocol Version No.**	**Date issued**	**Author(s) of changes**	**Details of Changes made**

Protocol amendments will be submitted to the REC and recorded in the Amendment History form.

PREVENT-PTSD

A randomised controlled trial to prevent PTSD, depression, and associated health problems in student paramedics (PREVENT-PTSD)

Statistical Analysis Plan

Version 1 – April 21, 2021

Based on Protocol version: Wild et al., 2018

Trial registration: ISRCTN16493616

Role	Name	Title	Signature	Date
Author	Esther Beierl	Dr.	Esther Beierl	29 July 2021
Senior Statistician	Susan Dutton	Assoc. Prof.	S. Dutton	16 August 2021
Chief Investigator	Jennifer Wild	Dr.	Jennifer Wild	29 July 2021

CONTENTS

1. INTRODUCTION 30
- 1.1 Key personnel 30
- 1.2 Changes from previous version of SAP 30

2. BACKGROUND AND OBJECTIVES 31
- 2.1 Background and rationale 31

NEUROBIOLOGICAL FACTORS LINKED TO PTSD AND MD 32
- 2.2 Objectives 34

3. STUDY METHODS 34
- 3.1 Trial Design/framework 34
- 3.2 Randomisation and Blinding 35
- 3.3 Sample Size 35
- 3.4 Statistical Interim Analysis, Data Review and Stopping guidelines 36
- 3.5 Timing of Final Analysis 36
- 3.6 Blinded analysis 37
- 3.7 Statistical Analysis Outline 37

4. STATISTICAL PRINCIPLES 38
- 4.1 Statistical Significance and Multiple Testing 38
- 4.2 Definition of Analysis Populations 39

5. TRIAL POPULATION AND DESCRIPTIVE ANALYSES 39
- 5.1 Representativeness of Study Sample and Patient Throughout 39
- 5.2 Withdrawal from treatment and/or follow-up 40
- 5.3 Baseline Comparability of Randomised Groups 40
- 5.4 Unblinding 40
- 5.5 Description of Compliance with Intervention 40
- 5.6 Reliability 41

6. ANALYSIS 41
- 6.1 Outcome Definitions 41
- 6.2 Analysis Methods 42
- 6.3 Missing Data 46
- 6.4 Sensitivity Analysis 46
- 6.5 Pre-specified Subgroup Analysis 47
- 6.6 Supplementary/ Additional Analyses and Outcomes 47
- 6.7 Harms 49
- 6.8 Health Economics and Cost Effectiveness 50
- 6.9 Meta-analyses (if applicable) 50

7. SPECIFICATION OF STATISTICAL PACKAGES 50

8. REFERENCES 51

INTRODUCTION

Key personnel

SAP author: Dr. Esther Beierl

Trial statistician: Dr. Esther Beierl

Chief investigator: Dr. Jennifer Wild

Reviewers (e.g, Trial Manager, DSMC, TSC, Statistician as appropriate): Associate Professor Susan Dutton

Approver (e.g., Senior Statistician, Chief Investigator): Associate Professor Susan Dutton, Dr Jennifer Wild

Changes from previous version of SAP

A summary of key changes from earlier versions of SAP, with particular relevance to protocol changes that have an impact on the design, definition, sample size, data quality/collection and analysis of the outcomes will be provided. Include protocol version number and date.

Version number<br>Issue date	Author of this issue	Protocol Version & Issue date	Significant changes from previous version together with reasons
V1_21042021		Protocol_Wildetal2018	Not applicable as this is the 1st issue

BACKGROUND AND OBJECTIVES

Background and rationale

Emergency workers are at risk for major depression (MD) and posttraumatic stress disorder (PTSD), and an associated risk of poor physical health due to the nature of their work.[1] To date, interventions aimed at reducing risk of mental ill health in this population have been unsuccessful. Randomised controlled trials (RCT) found that trauma risk management, a peer support system widely available to the police and ambulance services in England, [2] critical incident stress debriefing widely used by UK fire-services, [3] and the charity Mind's six-session group-based resilience intervention had no effect on resilience or rates of mental ill health [4]. More effective preventative interventions for emergency workers are urgently needed.

Established interventions may have been unsuccessful because they fail to target predictors of mental ill health and are offered to emergency workers after rather than before repeated exposure to the stresses linked to their work. Moreover, cognitive strategies that could help them cope with characteristic stressors are not included as part of the training. For example, our and others' research has demonstrated that exposure to trauma or stressful scenarios through imagery reduces anxiety for police officers and other at risk populations. [5, 6] Development of more effective interventions requires identification of predictors of mental disorders and an understanding of how to modify them.

In a series of experimental and prospective studies, we identified two cognitive factors that are robust predictors of poor mental health in emergency workers: rumination (repetitive negative thinking) and resilience appraisals. Those who reported ruminative thoughts during critical incidents were more likely to experience poor levels of coping. [7] Adaptive appraisals during analogue trauma led to more successful attempts to regulate

emotions and fewer PTSD symptoms. [8] Our large-scale prospective study of newly recruited paramedics investigated predictors of PTSD and MD derived from cognitive theories of PTSD and depression. [1] Rumination at the start of paramedic training uniquely predicted PTSD; low resilience uniquely predicted an episode of MD.

We then developed an intervention to modify peri-traumatic ruminative thinking (i.e, thinking repetitively in an abstract way during trauma). Training to think in a concrete style (e.g., focusing on objective details and the sequence of events) led to significantly fewer intrusive memories and PTSD symptoms than individuals trained in a ruminative style. [9] We also applied one of the core techniques of a successful treatment for PTSD (cognitive therapy for PTSD,[10]), updating the memory of the stressful event with helpful information, as a preventative strategy for dealing with analogue trauma and found that it was more helpful in reducing repetitive thinking and PTSD symptoms than control interventions including exposure. [11]

Research has further demonstrated that exposure to trauma or stressful scenarios through imagery reduces anxiety for police officers and other at risk populations, and that internet-based cognitive treatment that includes attention training as a core component significantly reduces anxiety. [5, 6, 12]

Neurobiological Factors linked to PTSD and MD

Genetic and longitudinal studies suggest that inflammation is a pre-existing vulnerability factor for the development of PTSD in trauma-exposed individuals rather than simply a correlate of subjective distress, disease severity, or maladaptive coping strategies following PTSD onset. [13, 14] For example, brain imaging studies have shown that high inflammation levels may increase threat perception (negative valence). Peripheral

administration of lipopolysaccharides (LPS), residues from bacterial cells' components known to elicit a strong systemic inflammatory response, potentiates amygdala activity in response to socially threatening stimuli (fear faces).[15] In turn, greater pre-treatment amygdala reactivity to threat predicts less symptom reduction during CBT.[16] Additionally, inflammation is an important risk factor for depression and cardiovascular disease, which frequently accompany PTSD.[17-19] Our study will investigate the link between inflammation and the development of PTSD and MD in trauma-exposed student paramedics.

We will investigate whether or not iCT-R can reduce levels of clinically-relevant inflammation levels, such as C-Reactive Protein (CRP), known to increase risk of psychiatric as well as cardiovascular and metabolic conditions comorbid with PTSD and MD.

Given the wealth of literature supporting a relationship between the stress hormone, cortisol, and PTSD and MD, we will also systematically assess the cortisol awakening response (CAR) and diurnal cycle. The CAR is an endocrine marker, defined as the change in cortisol concentration that occurs during the first hour after waking from sleep.[20] A meta-analysis of 62 studies concluded that increases in the CAR were associated with job stress and life stress and linked to greater fatigue, burnout and exhaustion and risk for later health states, such as coronary heart disease.[21] A recent study found that higher CAR predicted future episodes of MD within a 2.5-year period.[20] We anticipate that iCT-R will reduce the CAR and cortisol throughout the day and protect against the development of PTSD and MD.

Objectives

The primary trial objective is to evaluate whether the newly developed iCT-R intervention is more effective than the existing Mind-Online intervention at 1-year and at 2-years follow-up with regards to PTSD and MDD diagnoses.

Secondary trial objectives are to evaluate the efficacy of iCT-R compared to Mind-Online at 1-year and at 2-year follow-up with regards to secondary outcomes, such as resilience and well-being measures, as well as general health measures, and in terms of health economic costs. Furthermore, the effects of iCT-R versus Mind-Online on immune and hormone function will be compared at post-intervention.

Tertiary trial objectives are to investigate whether risk factors and predictors at baseline, such as level of social support, trauma exposure, demographic data, neuroticism as a personality factor, level of concrete thinking, frequency, duration, and distress with regards to intrusive memories influence any effects of iCT-R on PTSD and MDD diagnoses, psychological distress, and well-being at 1-year follow-up.

Further tertiary objectives are to investigate whether changes from baseline to post-intervention in resilience factors, such as rumination, responses to intrusive memories, level of concrete thinking, and resilience appraisals, predict PTSD and MDD diagnoses at 1-year follow-up in the iCT-R intervention group.

STUDY METHODS

Trial Design/framework

Interventions:

The study is a single-blind (assessors blind) randomised controlled trial. There are three arms: iCT-R, Mind-Online, and Standard Practice. The newly developed intervention iCT-R aims to modify rumination and appraisals linked to low resilience and risk for PTSD and MDD with a six-session supported online intervention. A trained research assistant provides email feedback on students’ responses and, through an automated SMS programme, sends regular reminders of key points of the intervention and notifications to practice the skills. The other intervention also contains six online modules and includes information and advice about stress, sleep problems, anger, depression, PTSD, and mindfulness. Participants will receive the same frequency, type and duration of online support as in iCT-R. The third

arm is Standard Practice. Participants have access to the usual support offered through their university, but they will not receive any online modules or online support.

Time points:

Data are collected pre-intervention, post-intervention, at 6-months follow-up, at 1-year follow-up and at 2-years follow-up.

More information on the intervention arms and measurement time points can be found in the published trial protocol (Wild et al., 2018).

Randomisation and Blinding

Participants are randomised on a 1:1:1 ratio as per a computer-generated randomisation schedule by OCTRU (Oxford Clinical Trials Research Unit) stratified by site, gender and baseline PHQ-9 score (≥ 9 vs. < 9) and PCL-5 score (≥ 33 vs. < 33). The researchers inform participants of the intervention they are to receive after participants have completed baseline assessments. Outcome assessment is single blind. Online questionnaires throughout the intervention and follow-up time points are completed without any involvement of the researchers. The clinical interview is done by an independent assessor, who is blind to treatment allocation. All personnel involved in the blood and saliva samples are blinded to intervention allocation. Due to the nature of the interventions, participants cannot be completely blinded to allocation.

Sample Size

There are no interventions for emergency workers which target modifiable risk factors. Power calculation was approximated using the results of a study with a similar approach. Topper et al. (2017) found that an intervention targeting rumination reduced rates of depression in adolescents at 1-year follow-up by 57% compared to wait-list. We estimated that iCT-R, which also aims to reduce rumination, would reduce PTSD and MDD diagnoses by 50% in comparison to Mind-Online which has shown no change in rates of PTSD or MDD over time based on previous research. Setting power at 80%, $\alpha = 0.05$ and hypothesising a reduction of relative risk of 50% results in an OR of 0.429 requires a total sample size of n = 304 to show a risk reduction of 50% between iCT-R and Mind-Online. Therefore, each intervention arm requires n = 152. Including the third treatment arm, which is

Standard Practice, the total sample size required is n = 456. Allowing for 20% attrition, a total sample size of N = 570 is required.

Statistical Interim Analysis, Data Review and Stopping guidelines

Registration and assessment data are captured online via Qualtrics. Participants are assigned a unique code for all data files and audio tapes. Access to the system is restricted to named study personnel and via password protection. The University of Oxford's (UO) IT services have arranged for the files to be encrypted and backed up on a weekly basis. The data are copied to separate tapes, that are inaccessible without the TSM database by the UO IT Services. Papers from clinical interviews are kept in locked cabinets at UO. The audiotapes from clinical interviews are backed up online with password-protection and access restricted to study personnel. The blood samples are centrifuged as soon as the laboratory at the University of Surrey receives them on the day of collection. Saliva samples are analysed at the University of Surrey by RIA analysis.

In line with the OCTRU and the Medicines for Human Use Clinical Trials Regulations (2004), we have not recruited a Data Monitoring Committee because recruitment and follow-up occur over a short period, there are minimal risks to participants and the trial protocol is not modified regardless of any interim data. No interim analyses are planned.

Timing of Final Analysis

Originally, as stated in the published trial protocol (Wild et al., 2018), we had planned to analyse the trial data once the trial was finished at two year follow-up. However, the outbreak of the Covid pandemic may have added additional risk to the mental health of emergency workers and therefore, if iCT-R leads to improvement in emergency workers' mental health compared to Mind-Online or Standard Treatment, it would be beneficial to roll out the intervention as soon as possible. It has been discussed and agreed with the Trial Steering Committee that data could be analysed early, namely after completion of the 1-year follow-up period, if rates of PTSD and MDD diagnoses in the total sample at 1-year follow-up are higher than expected. At 1 year follow-up, in the total sample, rates of PTSD are 8.7% compared to 8.3% in non-intervention studies of this population [1] and the general population rate of 3%. Rates of depression are 15.8% compared to 10.6% in non-intervention longitudinal studies of this population [1] and the general population rate of 10.3%. Since

there are higher than expected rates of PTSD and MDD diagnoses at 1-year follow-up in the total sample we now plan to analyse data at 1-year follow-up. Two-year follow-up data (including sub-thresholds) will be analysed by including them into the 1-year outcome models once they have been completed.

Blinded analysis

No blinded analyses are to take place prior to the final data lock currently planned.

Statistical Analysis Outline

Linear and logistic mixed effects modelling are used to test the primary and secondary trial objectives (with the exclusion of the health economic analyses, which will be conducted by Dr. Tsiachristas and therefore not part of this analysis plan, as well as the hormone and immune function analyses which will be conducted by Professors Carmine Pariante and Andrea Danese), which is the standard technique for analysing clinical trials.
For the primary and secondary trial objectives, 1-year follow-up is the short-term primary outcome time point (except for immune and hormone function for which the time-point is post-intervention), PTSD and MDD diagnoses are the primary outcome measures. Once completed, the 2-year PTSD and MDD diagnoses at 2-year follow-up are the long-term and main primary outcome time points.

Both repeated assessments and intervention arm are specified as categorical variables for reasons of flexibility in the modelling approach. Repeated assessments, intervention arm, their interaction effects, and baseline scores and covariates are estimated as fixed effects and random effects are estimated for time (level 1) nested within the participants (level 2). An unstructured variance-covariance matrix is used to model the error correlation structure within the participants.

Global comparisons for the main effect of intervention group and the interaction effects between time and intervention group are tested for primary and secondary trial objectives and further group comparisons are carried out if the global comparisons are significant. The hierarchy of the group comparisons reads as follows: 1) iCT-R versus Mind-Online, 2) iCT-R versus Standard Practice, and 3) Mind-Online versus Standard Practice. Site is introduced as a random effect in the analyses of the primary trial objectives. If non-significant, site will not be carried forward into further trial analyses.

Maximum Likelihood estimation is used. Assumptions for mixed effects modelling are tested and normality of residuals are confirmed for the outcome measures. If assumptions are violated, resampling approaches will be used.

Estimated/ adjusted mean differences and Cohen's d's are reported for continuous outcomes. Cohen's d's with standard deviation at baseline in the denominator are reported for the treatment effects from results of linear mixed effects models. Estimated/ adjusted odds ratios are reported for dichotomous outcomes together with absolute values. Confidence intervals are reported at the 95% level, two-sided.

Tertiary trial objectives to investigate any influences of baseline risk factors and predictors on iCT-R intervention effects on outcomes, such as PTSD or MDD diagnoses, psychological distress, or well-being, are tested using linear and logistic mixed effects models using the same analysis principles as for the primary and secondary trial objectives. The main effects of the respective baseline risk factors and predictors (time-invariant covariates) and the two-way interactions between each of those baseline predictors and time (categorical: post-intervention, 1-year follow-up; level 1) are analysed. Baseline scores of PTSD or MDD symptom severity, or psychological distress, or well-being are used as covariates.

In case of any significant interaction effects between time and baseline moderators, simple slope analyses are carried out to investigate the direction of any moderation effects. Further tertiary trial objectives to investigate whether changes in process measures, such as rumination, intrusive memories, and concrete thinking, from baseline to post-intervention in the iCT-R condition predict PTSD and MDD diagnoses at 1-year follow-up will be analysed using logistic regressions with difference scores from pre to post for the process measures and baseline scores of PTSD or MDD symptom severity as covariate.

CACE and sensitivity analyses are conducted for the primary trial objectives.

Results are reported in APA format.

STATISTICAL PRINCIPLES

Statistical Significance and Multiple Testing

In the case of different scaling, continuous variables are standardized for the analysis. Global comparisons are tested and if significant, group comparisons are then carried out.

If not specified otherwise, one or the other, main effects of intervention group or interaction effects between intervention group and time in favour of iCT-R, indicate intervention superiority.

All analyses will be carried out with a significance level of $p < .05$, two-sided.

No adjustments for multiple testing are made, as the primary trial objective is to evaluate any superiority in efficacy of the new resilience intervention compared to the existing online intervention (group comparison between these two groups of highest priority and directive hypothesis).

Confidence intervals are reported at the 95% level, two-sided.

Definition of Analysis Populations

The analysis population is intent-to-treat (ITT). All participants in the randomised groups are analysed. In addition, a complier-average causal effect analysis (CACE) will be conducted to test any influence of compliance on the intent-to-treat intervention effect of the primary trial objectives/ analyses. CACE is then compared with the intent-to-treat intervention effects resulting from the primary analyses and results are reported.

TRIAL POPULATION AND DESCRIPTIVE ANALYSES

Representativeness of Study Sample and Patient Throughout

Paramedic students aged 18 and above in the years 1, 2 or 3 of their paramedic training programme are eligible for the study. They are screened for levels of PTSD and MD, and a trained research assistant under supervision of the Principal Investigator will contact participants if they score in the clinical range on measures of PTSD or MD, or report suicidal ideation to evaluate whether they are eligible or need treatment. Participants will be excluded from the study if their symptoms are interfering with their lives and they would like treatment, and the research assistant will then offer information on evidence-based treatment in their local area.

The flow chart of participants through each stage of the trial, including numbers of participants randomly assigned, receiving the intervention, completing the study protocol, and analysed for the primary outcome is provided following current CONSORT guidelines.

Protocol violations or deviations and information relating to the screening data including potential numbers of ineligible patients randomised, together with reasons, are also described.

Withdrawal from treatment and/or follow-up

Number and percentage of participants who have withdrawn from the trial intervention will be summarised across intervention arms and for each intervention arm, together with reasons for withdrawal if available.

Numbers and percentage of participants lost to follow-up across intervention arms and within each intervention arm are also reported.

In addition, numbers of participants who have withdrawn or have been lost to follow-up are reported in the CONSORT flow chart.

Baseline Comparability of Randomised Groups

Total sample size and group sizes (iCT-R, Mind-Online, Standard Practice), means, standard deviations, frequencies at baseline for the groups and the total sample, and occurrence of missing data and loss to follow-up for the groups, for any compliance groups, and the total sample are reported.

Unblinding

All cases unblinded to blinded assessors are reported and summarised (absolute numbers and percentages).

Description of Compliance with Intervention

Level of compliance will be defined as 1) number of online modules completed during the intervention, 2) practice of tools.

A complier-average causal effect analysis (CACE) is used to test any influence of compliance on the intent-to-treat intervention effect of the primary trial objective analyses.

Reliability

The primary outcome measures, such as Structured Interviews by clinicians and self-reports for the primary outcomes and nearly all of the secondary outcome measures are standard instruments that have been validated and have shown good reliability.

Scoring is done as described in the manuals or publications for the measures and relevant sub-scales and Cronbach's Alphas are reported for the study sample.

Interrater-reliability is reported for 1-year follow-up data. 10% of diagnoses at 1-year follow-up are rated by a second rater.

To ensure consistency in the data, validation checks on the data are carried out before locking the dataset and analysis of the data. These include checking for potential data entry mistakes, duplicate records, checking the range of variable values, missing data value codes, etc.

ANALYSIS

Outcome Definitions

Outcome measures:

The primary outcome measure for the primary trial objective is PTSD and MDD diagnoses (SCID-5;). Secondary outcomes with regards to the primary trial objective are PTSD symptom severity (PCL-5) and MDD symptom severity (PHQ-9).

Outcome measures for the secondary trial objectives include resilience measures (Wagnild-Resilience Scale and CD-RISC, both continuous), well-being measures (subscale rumination of the RIQ, GAD-7, GHQ-12, WEMWBS), general health measures (weight and height; Insomnia severity index; Smoking Behaviour Questionnaire, sum score for both items; Alcohol Use Questionnaire, unit increase/ decrease/ stayed the same), health economic costs (intervention costs per QALY gained based on 4 measures), and hormone and immune function (collected with saliva samples and CRP plasma).

Measures to test the tertiary trial objective are risk factors and predictors at baseline, such as level of social support (adapted from a brief measure of social support), trauma exposure (trauma screener), demographic data (general information questionnaire), neuroticism as a personality factor (subscale neuroticism from the Eysenck Personality Questionnaire), level of concrete thinking (CAT), and frequency, duration, and distress with regards to intrusive memories (Intrusions Questionnaire).

More information on the outcome measures can be found in the published trial protocol (Wild et al., 2018).

Time points:

Data are collected at pre-intervention, post-intervention, at 6-months follow-up, at 1-year follow-up and at 2-years follow-up. PTSD and MDD diagnoses are assessed pre-intervention, post-intervention, at 1-year and at 2-years follow-up, PTSD and MDD symptom severity are assessed at all measurement points. Secondary outcome measures are assessed at pre-intervention, post-intervention, and at 1-year and at 2-years follow-up. All measures for the tertiary trial objective are assessed at pre-intervention, at 1-year and at 2-years follow-up. All tertiary measures (except for demographics) are assessed at post-intervention, and trauma exposure, concrete thinking and intrusion measures are collected at 6-months follow-up, as well.

For the early analysis of the trial results, 1-year follow-up is the short-term primary outcome time point. Once the trial has been completed, the primary outcome time point is 2-years follow-up as described in the published trial protocol.

Analysis Methods

Analyses of the primary trial objectives:

To analyse any differences between the intervention arms in terms of effectiveness with regards to rates of PTSD and MDD diagnoses (SCID-5) at 1-year follow-up (short-term primary outcome time point) and at 2-years follow-up (long-term primary outcome time point once completed), intervention condition and the interaction between intervention time and

intervention group on rates of PTSD and MDD diagnoses, logistic mixed effects models with PTSD and MDD diagnoses as outcomes (short-term outcome 1-year follow-up data, long-term outcome 2-years follow-up once completed) are specified and analysed.

To analyse any differences between the intervention arms in terms of effectiveness with regards to PTSD symptom severity (PCL-5) or MDD symptom severity (PHQ-9) at 1-year follow-up (short-term primary outcome time point) and at 2-years follow-up (long-term primary outcome time point once completed), intervention condition (categorical; iCT-R versus Mind-Online versus Standard practice; level 2) and the interaction between intervention time (categorical; post-intervention, 6-months follow-up; 1-year-follow-up, 2-year follow-up, once completed; level 1) and intervention group on PTSD or MDD symptom severity, linear mixed effects models with either PTSD or MDD symptom severity as outcomes are specified and analysed.

Both repeated assessments and intervention arm are specified as categorical variables for reasons of flexibility in the modelling approach. Repeated assessments/ time, intervention arm, both their interaction effects, and baseline scores and covariates are estimated as fixed effects and random effects are estimated for time (level 1) nested within the participants (level 2). An unstructured variance-covariance matrix is used to model the error correlation structure within the participants. Estimation method is Maximum Likelihood. Normality of the residuals for the outcomes is confirmed visually.

Randomisation has been done by stratification by site, gender, and baseline PTSD symptom severity and MDD symptom severity. Therefore, baseline PTSD symptom severity and MDD symptom severity and randomisation factors are added as covariates in these models. Site is added as random factor in those models. If non-significant, they will not be carried forward into further analyses.

In case of a significant main effect of intervention group or interaction effect between intervention group and time with regards to the primary outcome measure (PTSD and MDD diagnoses) and secondary outcome measures for the primary trial objective (PTSD and MDD symptom severity), intervention group comparisons are carried out using pairwise contrasts. iCT-R (coded as ‘1’) is tested against Mind-Online (coded as ‘0’) and a significant main effect or interaction in favour of iCT-R indicates intervention superiority (comparison of highest priority) of the newly developed resilience intervention over the existing online intervention. In a second step, iCT-R (coded as ‘1’) is then tested against Standard Practice

(coded as ‘0’) and again, a significant main effect or interaction in favour of iCT-R indicates intervention superiority for the newly developed intervention over usual Standard Practice. In a third step, Mind-Online (coded as ‘1’) is tested against Standard Practice (coded as ‘0’) and a significant main effect or interaction in favour of Mind-Online indicates intervention superiority of the existing online intervention compared to Standard Practice.
Adjusted/ estimated mean differences and Cohen’s d’s with standard deviation at baseline in the denominator are reported in tables for continuous outcomes for the intervention effects from results of linear mixed effects models and frequencies of diagnoses (absolute numbers and percentages) and absolute values as well as estimated/ adjusted odds ratios are reported for dichotomous outcomes.

All significance tests are carried out on a $p < .05$ level, two-sided, and confidence intervals are reported on the 95% level.

Analyses of secondary trial objectives:

Any intervention effects on the secondary outcome measures, such as resilience (Connor-Davidson Resilience Scale,; Wagnild Resilience Scale), rumination (ruminative responses brooding subscale; Responses to Intrusions Questionnaire rumination subscale), anxiety (GAD-7), Smoking Behaviour (Smoking Behaviour Questionnaire), Alcohol consumption (Alcohol Use Questionnaire), psychological distress (GHQ), well-being (Warwick Edinburgh Mental Wellbeing Scale), sleep quality (Insomnia severity index), and weight, are analysed for the short-term primary outcome time point of 1-year and for the long-term primary outcome time point of 2-years follow-up, once completed.

Linear mixed effects models are specified for these continuous outcome measures. Intervention condition (categorical; iCT-R versus Mind-Online versus Standard Practice; level 2) and the interaction between intervention time (categorical; post-intervention, 1-year-follow-up, 2-years-follow-up once completed; level 1) are specified as fixed effects predictors in the models.

Baseline values for the respective secondary outcome are added as covariate in these models together with PTSD and MDD symptom severity at baseline, and randomisation factors.

Adjusted/ estimated mean differences and Cohen's d's with standard deviation at baseline in the denominator are reported in tables for continuous outcomes for the intervention effects from results of linear mixed effects models.

The health economic analyses will be conducted and reported by Dr. Tsiachristas and and hormone and immune function are tested by collaborators and are therefore not part of this statistical analysis plan. A plan for the health economic and hormone and immune function analyses will be agreed.

Analyses of the tertiary trial objectives:

In order to investigate whether risk factors and predictors at baseline, such as level of social support, trauma exposure, demographic data, past and current comorbidities, neuroticism as a personality factor, level of concrete thinking, and frequency/ duration/ distress with regards to intrusive memories, influence any iCT-R intervention effects on PTSD or MDD diagnoses, psychological distress, and well-being at 1-year follow-up, a mixed effects model for each of the outcomes, PTSD and MDD diagnoses, psychological distress, and well-being, is specified with baseline scores of PTSD or MDD symptom severity or psychological distress or well-being as covariate. The main effects of the respective baseline predictors (time-invariant covariates) and the two-way interactions between each of those baseline predictors and time (categorical: post-intervention, 1-year follow-up; level 1) will be analysed.

Again, Maximum Likelihood is used if the assumption of normality of the residuals of the outcomes is fulfilled. An unstructured variance-covariance structure matrix is used, all significance tests are carried out on a $p < .05$ level, two-sided, and confidence intervals are reported at the 95% level.

In case of any significant interaction effects between time and baseline moderators, simple slope analyses are carried out to investigate the direction of any moderation effects.

Further tertiary trial objectives to investigate whether changes (decrease) in process measures, such as rumination, intrusive memories, and increases in concrete thinking, from baseline to post-intervention in the iCT-R condition predict lack of PTSD or MDD diagnoses

(versus presence of PTSD or MDD diagnoses) at 1-year follow-up are analysed using logistic regressions with difference scores from pre to post for the process measures and baseline scores of PTSD or MDD symptom severity as covariate.

CACE analysis:

A complier-average causal effect analysis (CACE) is used to test the influence of compliance on the intent-to-treat intervention effect of the primary trial objectives/ analyses. Magnitude of compliance will be defined as 1) number of online modules completed during the intervention, and 2) practice of tools. Based on these variables, trajectories of compliance are determined. CACE will then be compared with the intent-to-treat intervention effects resulting from the primary analyses and the results will be reported.

Missing Data

Missing data mechanisms are explored and reported.

Missing data across and between intervention arms are reported and compared.

The RCT is intent-to-treat from randomisation. All randomised cases with any missing data will be included in the analyses of PTSD and MDD diagnoses and secondary outcomes (linear mixed effects models are considered to be reasonably robust to missing data at random).

Sensitivity analyses for the primary trial objectives will be carried out to test robustness of the results to any missing data mechanism.

Sensitivity Analysis

Sensitivity analyses will be carried out for the analyses of the primary trial objectives. We will test whether any intervention superiority or non-inferiority with regards to PTSD and MDD diagnoses is robust/ holds when 1) analysing data including cases with missing data, 2)

analysing data including any potential covariates that are predictive of any missing data, and 3) imputation.

Pre-specified Subgroup Analysis

Intervention effects of iCT-R on PTSD and MDD diagnoses at 1-year follow-up (short-term primary outcome time point) and at 2-year follow-up (once completed, long-term primary outcome time point)are presented using forest plots for participants who had higher versus lower PTSD or MDD symptom severity at baseline.

Supplementary/ Additional Analyses and Outcomes

Additional exploratory analyses due to the Covid pandemic, suggestions taken from FDA guidelines (June 2020) [22] and Mayer et al. (2020) [23] and adapted to the needs of the current trial:

The trial took place before and during the outbreak of the Covid pandemic. Additional reporting of descriptive statistics and missing data, additional exploratory analyses, and sensitivity analyses are needed to capture any possible impact of the pandemic on the main trial objectives.

Two Covid periods (pre-Covid/ before March 2020, which marks the first UK national lockdown, or possibly after-Covid, coded as ‘0’, and during-Covid, since March 16, 2020, coded as ‘1’) are defined. For each participant at each assessment time point it will be classified whether the assessment took place before or after Covid impact versus during Covid impact. This way exogeneous time-varying covariates are generated for further analyses.

Additionally, a COVID questionnaire was implemented to capture any possible subjective impact by COVID reported by the participants on outcome.

Main trial analyses and Covid:

Descriptive statistics for the two outcome measures for the primary trial objectives (PTSD and MDD diagnoses and PTSD or MDD symptom severity) are reported for the whole sample and by intervention groups based on the Covid grouping variables for the short-term primary outcome time point at 1-year follow-up and the 2-year follow-up assessment point, once completed (means and standard deviations for continuous outcomes and absolute numbers and percentages for diagnoses).

In the case of any differences, additional analyses are carried out to explore whether there is any possible intervention heterogeneity depending on participants' Covid status on PTSD and MDD diagnoses or PTSD and MDD symptom severity at 1-year follow-up or at 2-year follow-up, once completed. In this case, linear and logistic mixed models are specified in the same way as proposed for the analyses of the primary trial objectives and participants' Covid status variables are entered as exogeneous time-varying covariates. Two-way interaction terms between those covariates and intervention group as well as three-way interaction terms between Covid status, time, and intervention group are analysed.
In the case of significant interaction terms, which could possibly indicate intervention heterogeneity, additional effect size measures are reported. Adjusted mean differences and Cohen's d's with standard deviation at baseline in the denominator are reported in tables for continuous outcomes from results of linear mixed effects models and frequencies of diagnoses (absolute numbers and percentages) and absolute differences as well as odds ratios are reported for dichotomous outcomes.

In addition to Covid status depending on time points, the COVID questionnaire, scored as a continuous variable, which assesses the self-reported impact of COVID on a participant will be descriptively reported and analysed as a covariate in a similar way like the time-based covariates to determine whether or not it influences outcome. We will compare rates of PTSD and MDD for the sample who score positive on the item ('I am graduating

early as a result to support the NHS workforce') compared to participants who do not endorse this item.

Exploration of missing data patterns and possibly additional sensitivity analyses:

Additional missing data explorations are reported. Any missing data, withdrawal from the intervention, or loss to follow-up are reported in tables for the whole sample and by intervention groups based on the Covid status variables for the short-term primary outcome time point 1-year follow-up and the 2-year follow-up assessment point, once completed (means and standard deviations for continuous outcomes, absolute numbers and percentages for dichotomous outcomes).

In the case of any missing data, withdrawal, or loss to follow-up dependent on Covid status or the endorsement of the COVID questions, additional sensitivity analyses need to be carried out.

CACE analyses:

In the case of potential intervention heterogeneity due to any influence of the Covid pandemic, additional CACE analyses are carried out, as well, to test whether Covid status or questions had an influence on compliance to the interventions. In case of any differences the Covid CACE is compared with the intent-to-treat intervention effects resulting from the primary trial analyses and results are reported.

Harms

No adverse events are anticipated. The procedure for adverse events (AE) and serious adverse events (SAE) are reported in the trial protocol paper.

The total number of serious adverse events (SAE) and the number of SAE per participant (if at least one SAE) are reported in each randomised intervention group. If there is at least one SAE, a comparison between the intervention groups regarding the total number of SAE and the number of SAE per participant is made. The safety analysis is also ITT. Results of these comparisons, such as statistical tests and ratios, are reported.

No additional safety analyses are needed due to the Covid pandemic as the continuation of the trial during the pandemic did not put participants at a higher risk.

Health Economics and Cost Effectiveness

Health economic analyses (intervention costs per QALY gained) will be conducted by Dr. Apostolos Tsiachristas and are therefore not part of this analysis plan.

Meta-analyses (if applicable)

No meta-analyses are planned.

SPECIFICATION OF STATISTICAL PACKAGES

All analyses will be done in RStudio [24]. The main trial objectives are analysed using the R packages *lme4* [25] and *nlme* [26].

REFERENCES

1 Wild J, Smith KV, Thompson E, et al. A prospective study of pre-trauma risk factors or posttraumatic stress disorder and depression. *Psychol Med* 2016;46(1):2571-82.

2 Greenberg N, Langston V, Everitt, B., Iversen, A., Fear, N.T., Jones, N. & Wessely, S. (2010). A cluster randomised controlled trial to determine the efficacy of trauma risk management (TRiM) in a military population. *J Trauma Stress, 23,* 430-36.

3 van Emmerik AA, Kamphuis JH, Hulsbosch AM, Emmelkamp PM (2002). Single session debriefing after psychological trauma: a meta-analysis. *Lancet* 2002;360:766-71.

4 Wild, J. (2016). An evaluation of Mind's resilience intervention for emergency workers. Final Report.

5 Arnetz BB, Arble E, Backman L, Lynch A, & Lublin A. Assessment of a prevention program for work-related stress among urban police officers. *Int Arch Occup Environ Health* 2013;86:79-88.

6 Wild J, Hackmann A, Clark DM. Rescripting early memories linked to negative images in social phobia: A pilot study. *Behav Ther* 2008;39:47-56.

7 Shepherd L, Wild J. Cognitive appraisals, objectivity, and coping in ambulance workers: a pilot study. *Emerg Med J* 2013;31:41-4.

8 Shepherd L, Wild J. Emotion regulation, physiological arousal and PTSD symptoms in trauma-exposed individuals. *J Behav Ther Exp Psychiatry* 2014;45:360-7.

9 White R, Wild J. "Why" or "How": The effect of concrete versus abstract processing on intrusive memories following analogue trauma. *Behav Ther* 2016:47(3):404-415.

10 Ehlers A., Hackmann A., Grey N, Wild J, et al. A randomized controlled trial of 7-day intensive and standard weekly cognitive therapy for PTSD and emotion-focused supportive therapy. *Am J Psychiatry,* 2014:171, 294-304.

11 Pile V, Barnhofer T, Wild J. Updating versus exposure to prevent consolidation of conditioned fear. *PLoS One* 2015;10:1-21.

12 Stott R, Wild J, Grey N et al. Internet-delivered cognitive therapy for social anxiety disorder: a development series. *Behav Cogn Psychother* 2014;41:383-97.

13 Michopoulos V, Rothbaum AO, Jovanovic T, et al. Association of CRP genetic variation and CRP level with elevated PTSD symptoms and physiological responses in a civilian population with high levels of trauma. *Am J Psychiatry,* 2015;172:353-362.

14 Eraly SA, Nievergelt CM, Maihofer AX, et al. Assessment of plasma C-reactive protein as a biomarker of posttraumatic stress disorder risk. *JAMA Psychiatry* 2014;71:423-431.

15 Inagaki TK, Keely A, Muscatell MA, et al. Inflammation Selectively Enhances Amygdala Activity to Socially Threatening Images. *Neuroimage 2012;*15:3222-6.

16 Bryant RA, Felmingham K, Kemp A, et al. Amygdala and ventral anterior cingulate activation predicts treatment response to cognitive behaviour therapy for post-traumatic stress disorder. *Psychol Med* 2008;*38:*555-61.

17 Miller AH, Raison CL. The role of inflammation in depression: from evolutionary imperative to modern treatment target. *Nat Rev in Immunol* 2016;16:22-34.

18 Ridker PM, Cushman M, Stampfer MJ, et al. Inflammation, Aspirin, and the Risk of Cardiovascular Disease in Apparently Healthy Men. *N Engl J Med* 1997;336:973-9.

19 Sumner JA, Kubzansky LD, Elkind MS, et al. Trauma Exposure and Posttraumatic Stress Disorder Symptoms Predict Onset of Cardiovascular Events in Women. *Circulation* 2015;8:251-9.

20 Vrshek-Schallhorn S, Doane LD, Mineka S, et al. The cortisol awakening response predicts major depression: predictive stability over a 4-year follow-up and effect of depression history. *Psychol Med* 2013;43:483-93.

21 Chida Y, Steptoe A. Cortisol awakening response and psychosocial factors: a systematic review and meta-analysis. *Biol Psychiatry* 2009;80:265-78.

22 FDA (2020, June). Statistical Considerations for Clinical Trials During the COVID-19 Public Health Emergency: Guidance for Industry. Retrieved from https://www.fda.gov/media/139145/download.

23 Mayer, R D, et al. Statistical issues and recommendations for clinical trials conducted during the COVID-19 pandemic. *Statistics in Biopharmaceutical Research* 2020;12:399-411.

24 RStudio Team. *RStudio: Integrated development for R*, 2021. Boston, MA: RStudio, Inc.

25 Bates D et al. Fitting linear mixed effects models using lme4. *Journal of Statistical Software 2015;67*:1-48.

26 Pinheiro J et al. *nlme: Linear and nonlinear mixed effects models* 2020. https://CRAN.R-project.org/package=nlme
