# Supplementary material for: Cognitive Resilience Training to Prevent PTSD and Major Depressive Disorder in Paramedic Recruits: A Randomized Clinical Trial
Source: JAMA Netw Open. 2026 Feb 9;9(2):e2557241. doi: 10.1001/jamanetworkopen.2025.57241 (PMC12887744; doi:10.1001/jamanetworkopen.2025.57241)
Supplement: Supplement 2. — eAppendix 1. Description of Interventions eTable 1. Brief TiDiER Comparison Table: Internet-Delivered Cognitive Training in Resilience (iCT-R) vs Psychoeducation (Edu) eTable 2. Description of Modules by Condition eAppendix 2. Tertiary Outcomes eTable 3. Means and Standard Deviations or N (Percent) of Tertiary Outcome Measures at Each Time Point eTable 4. Rates of Participants Achieving Completion, Compliance and Minimum Dose Per Condition eTable 5. Rates of Completion Per Module and Time Spent on Modules by Intervention Condition eAppendix 3. Sensitivity, CACE and Mediation Analyses eTable 6. Sensitivity Analysis: Primary Analysis With the Three Baseline Clinical Cases Removed eTable 7. Sensitivity Analyses (Minimal Compliance and Core Module Completion): Odds of Meeting Criteria for PTSD and MDD at Follow-Up eTable 8. Mediation of 12-Month Scores on the PCL-5 and PHQ-9 [file jamanetwopen-e2557241-s002.pdf]

Supplemental Online Content

Wild J, Tyson G, Thew G. Cognitive resilience training to prevent PTSD and major depressive disorder in paramedic recruits: a randomized clinical trial. *JAMA Netw. Open*. 2026;9(2):e2557241. doi:10.1001/jamanetworkopen.2025.57241

eAppendix 1. Description of Interventions
eTable 1. Brief TiDiER Comparison Table: Internet-Delivered Cognitive Training in Resilience (iCT-R) vs Psychoducation (Edu)
eTable 2. Description of Modules by Condition
eAppendix 2. Tertiary Outcomes
eTable 3. Means and Standard Deviations or N (Percent) of Tertiary Outcome Measures at Each Time Point
eTable 4. Rates of Participants Achieving Completion, Compliance and Minimum Dose Per Condition
eTable 5. Rates of Completion Per Module and Time Spent on Modules by Intervention Con-dition
eAppendix 3. Sensitivity, CACE and Mediation Analyses
eTable 6. Sensitivity Analysis: Primary Analysis With the Three Baseline Clinical Cases Removed
eTable 7. Sensitivity Analyses (Minimal Compliance and Core Module Completion): Odds of Meeting Criteria for PTSD and MDD at Follow-Up
eTable 8. Mediation of 12-Month Scores on the PCL-5 and PHQ-9

This supplemental material has been provided by the authors to give readers additional information about their work.

© 2026 Wild J et al. *JAMA Network Open*.

eAppendix 1. Description of Interventions

Internet-delivered cognitive training in resilience

Internet-delivered cognitive training in resilience (iCT-R) is a six-module programme delivered weekly over six weeks. The programme adopts the active, experiential approach of internet-delivered cognitive therapy interventions, incorporating strategies from internet-delivered cognitive therapy for PTSD (iCT-PTSD; Ehlers et al., 2023), social anxiety disorder (iCT-SAD; Clark et al., 2023) and rumination-focused cognitive therapy for depression (Watkins et al., 2011). Four modules target modifiable risk factors for PTSD and MDD identified in prospective research: negative appraisals related to low resilience (Module 2) (Wild et al., 2016), repetitive negative thinking as rumination and worry (Modules 3 and 5) (Topper et al., 2017; Wild et al., 2016), and the 'here and now' quality of intrusive memories (Module 4) (Halligan et al., 2003). Following consultation with PPI contributors, two additional modules were included: one targeting self-focused attention to reduce self-consciousness while working under observation, and another on managing emerging PTSD and depression symptoms.

Psychoeducation

The comparison intervention consisted of six weekly online psychoeducation modules covering sleep, stress, depression, anger, mindfulness, and PTSD, respectively. Content available on the Mind website (a UK mental health charity) was adapted for emergency workers and previously evaluated (Wild, El-Salahi, Degli Esposti, et al., 2020).

eTable 1. Brief TiDiER comparison table: Internet-delivered cognitive training in resilience (iCT-R) vs psychoducation (Edu)

TiDiER Item	iCT-R	Edu
**Brief name**	Internet-delivered Cognitive Therapy for Resilience (iCT-R)	Psychoeducation (Edu)
**Mode of delivery**	Online, supported by wellbeing coach with undergraduate qualification in psychology	Online, supported by wellbeing coach with undergraduate qualification in psychology
**Theoretical Rationale**	Evidence-based cognitive therapy techniques to target modifiable risk factors	Psychoeducation approach covering specific mental health topics relevant to high risk occupations
**Materials**	Online modules with videos and interactive exercises	Online modules with video
**Duration**	6 weeks (1 module per week)	6 weeks (1 module per week)
**Setting**	Online platform	Online platform

© 2026 Wild J et al. *JAMA Network Open*.

eTable 2. Description of Modules by Condition

Week	iCT-R	Edu
**1**	**Module:** It Matters What you Focus On: Helpful and Unhelpful Attention	**Module:** Stress
	**Primary Focus:** Self-focused attention<br>**Intervention exercises:** Two student paramedics explain how externally-focused attention helps them, behavioural experiment to test the effects of shifting focus of attention, Attention Gym with 2 x 5 mins attention training exercises.<br>**Tools:** (1) Behavioural experiment to test predictions associated with feeling self-conscious; (2) 4 steps to shift from self-to-externally focused attention	**Primary Focus:** Understanding and managing stress<br>**Psychoeducation:** Identifying signs of stress, identifying and managing pressure, identifying triggers for stress, strategies for organising time, how to address the causes of stress, how to accept situations that cannot change.<br>**Tools:** (1) Steps to spot signs for stress, (2) Strategies for organising time.
**2**	**Module:** Get Out of Your Head with Helpful Thinking	**Module:** Sleep
	**Primary Focus:** Updating negative appraisals related to low resilience<br>**Intervention exercises:** Identifying extreme thinking, exercise to update extreme thoughts linked to low resilience (negative self-beliefs), exercise to elicit the effects of flexible and extreme thinking, audio exercise to elicit the effects of abstract vs practical thinking, video exercise applying practical thinking to critical incidents.<br>**Tools:** (1) 3 steps to elicit practical thinking, (2) Behavioural experiment to update negative self-belief(s)	**Primary Focus:** Sleep-mental health connection<br>**Psychoeducation:** Effect of poor sleep on mental health, sleep improvement strategies, breathing exercises for relaxation, shift work and sleep management, sleep diary, challenging unhelpful thought patterns, sleep checklist of strategies to implement.<br>**Tools:** (1) Breathing exercise for relaxation, (2) sleep diary to identify causes of poor sleep
**3**	**Module:** Habits and Dwelling: How to Change Them	**Module:** Depression
	**Primary Focus:** Rumination<br>**Intervention exercises:** Identifying dwelling with 3 questions, video illustration of how habits develop (through associative learning), video testimony of a student paramedic describing his warning signs for dwelling, exercise to elicit IF-THEN plan to apply to warning signs for rumination.<br>**Tool:** (1) IF-THEN plan	**Primary Focus:** Understanding and managing depression<br>**Psychoeducation:** Depression symptoms and causes, breaking negativity cycles, strategies to stay active, how to improve social connection, practice self-care, applying exercise to low mood, supporting friends with depression. Video of 5 people with lived experience and how they coped with depression.<br>**Tool:** (1) Breaking the cycle of negativity

© 2026 Wild J et al. *JAMA Network Open*.

**4**	**Module:** Dealing with Unwanted Memories: Then vs Now	**Module:** Anger
	**Primary Focus:** Intrusive memories (including rumination as a response)<br>**Intervention exercises:** Video testimony (how 2 student paramedics deal with unwanted memories), video exercise to elicit the effects of suppression, how to use Then vs Now to triggers in the present that overlap with stimuli from past trauma or critical incidents, practice exercise with video clips and audio triggers, responsibility pie chart for guilt/self-blame, managing low mood linked to memories.<br>**Tools:** (1) Then vs Now (2) Responsibility pie chart for guilt	**Primary Focus:** Understanding and controlling anger **Psychoeducation:** Understanding when anger is a problem, anger management strategies, identifying triggers for anger, spotting warning signs, calming techniques, assertiveness skills, supporting friends with anger issues.<br>**Tools:** (1) Breathing slowly (2) Relaxation techniques
**5**	**Module:** Transforming Worries and Improving Performance	**Module:** Mindfulness
	**Primary Focus:** Worry<br>**Intervention exercises:** Video testimony (how 2 student paramedics deal with worries), worry diary to determine the percentage of worries that come true, calculating realistic risk for worries, behavioural experiment to test worry predictions, imagery exercise to focus on the likely (rather than catastrophic) outcome, planning ahead exercise.<br>**Tools:** (1) Worry diary (2) Realistic risk and (3) Planning ahead.	**Primary Focus:** Mindfulness techniques and relaxation<br>**Psychoeducation:** How to practice mindfulness, suggested mindful walking exercise, general relaxation methods, suggested body relaxation exercise, suggested creativity exercise.<br>**Tools:** (1) Mindful walking (2) Body relaxation (3) Creativity exercise.
**6**	**Module:** Beating Stress and Trauma: My Blueprint	**Module:** PTSD
	**Primary Focus:** Responding to emerging symptoms of PTSD and depression<br>**Intervention exercises:** Interactive true/false quiz with feedback and key points from modules. PTSD Guide with checklist of symptoms and summary of specific strategies for unwanted memories, dwelling, avoidance, unhelpful thoughts, difficult feelings, including anger, and sleep problems. Depression Guide with weekly activity planner, and	**Primary Focus:** PTSD awareness and support<br>**Psychoeducation:** Understanding PTSD, seeking support from other people, treatment options, peer support techniques for helping someone with PTSD.<br>**Tools:** (1) Peer support technique for trauma exposure

© 2026 Wild J et al. *JAMA Network Open*.

	practical strategies to apply to maintaining beliefs and behaviours.<br>**Tools:** (1) Then vs Now for unwanted memories (2) IF-THEN for rumination (3) Depression activity planner	

© 2026 Wild J et al. *JAMA Network Open*.

eAppendix 2. Tertiary Outcomes

Potential Moderators

Tertiary outcomes assessed trauma exposure with the Life Events Checklist (Gray et al., 2004), neuroticism with the neuroticism subscale of the Eysenck Personality Questionnaire (Eysenck & Eysenck, S.B.G, 1975), perceived social support from friends, family and work colleagues with a brief measure of social support (Sarason et al., 1987), concrete thinking with the Concrete and Abstract Thinking questionnaire (Lorenz et al., 2025), and practice of intervention tools with a single item question asking participants whether they practiced tools from the training. The latter question was administered only to participants who received iCT-R or the psychoeducation intervention.

Health Outcomes

Participants reported their weekly average alcohol consumption in units and, for smokers, the average number of cigarettes smoked per day. Weight was self-reported in kilograms, and sleep problems were assessed using the Insomnia Severity Index (Morin et al., 2011). Results are reported in the manuscript in Table 4. Health-related quality of life was measured with the five-level version of the EuroQol-5 Dimensions questionnaire (EuroQuol Research Foundation, n.d.), which forms part of the health economics evaluation. In accordance with the statistical analysis plan, health economics outcomes are reported separately.

© 2026 Wild J et al. *JAMA Network Open*.

eTable 3: Means and standard deviations or N (percent) of tertiary outcome measures at each time point

Tertiary Outcomes		iCT-R	Edu	Standard	Total
Trauma history (LEC-5)					
	Baseline	2.96 (2.33) [195]	2.81 (2.49) [197]	3.46 (3.05) [178]	3.06 (2.64) [570]
	Post	0.45 (0.87) [169]	0.34 (0.68) [179]	0.37 (0.84) [164]	0.38 (0.80) [512]
	12m	2.25 (2.17) [158]	2.61 (2.16) [165]	2.51 (2.07) [152]	2.45 (2.14) [475]
Neuroticism (EPQ)					
	Pre	19.11 (3.04) [195]	18.77 (3.07) [197]	19.22 (3.01) [178]	19.03 (3.04) [570]
	Post	19.79 (3.01) [169]	19.79 (3.22) [179]	19.64 (2.95) [164]	19.74 (3.06) [512]
	12m	19.93 (3.05) [158]	19.08 (3.12) [165]	19.81 (2.96) [152]	19.60 (3.06) [475]
Social support: Friends & Family (SS-FF)					
	Pre	38.90 (6.89) [195]	38.78 (6.82) [197]	37.90 (6.92) [178]	38.55 (6.88) [570]
	Post	40.01 (6.10) [169]	39.04 (7.22) [179]	38.87 (7.54) [164]	39.30 (6.98) [512]
	12m	39.35 (7.21) [158]	38.64 (7.60) [165]	39.19 (6.63) [152]	39.05 (7.16) [475]
Social support: Work (SS-W)					
	Pre	27.11 (6.05) [195]	27.89 (6.22) [197]	27.03 (6.28) [178]	27.36 (6.18) [570]
	Post	28.44 (6.48) [169]	28.09 (6.91) [179]	26.98 (6.37) [164]	27.85 (6.61) [512]
	12m	26.87 (6.81) [158]	27.71 (6.71) [165]	26.34 (6.69) [152]	26.99 (6.75) [475]
Concrete Thinking (CAT)					
	Pre	56.45 (18.47) [195]	60.24 (17.91) [197]	59.55 (20.37) [178]	58.73 (18.94) [570]
	Post	68.66 (18.60) [169]	69.64 (18.28) [179]	67.02 (16.64) [164]	68.48 (17.88) [512]
	6m	70.99 (17.70) [147]	69.42 (15.82) [166]	67.93 (18.03) [146]	69.45 (17.16) [459]
	12m	70.74 (16.25) [158]	69.07 (15.13) [165]	69.50 (16.09) [152]	69.76 (15.80) [475]
Practice of Tools (N/%)					
Post	Practiced	164 (97.6) [168]	105 (59.0) [178]	Not assessed	269 (77.7) [346]
	Did not practice	4 (2.4) [168]	73 (41.0) [178]	-	77 (22.3) [346]
6m	Practiced	136 (93.8) [145]	93 (55.7) [167]	-	229 (73.4) [312]
	Did not practice	9 (6.2) [145]	74 (44.3) [167]	-	83 (26.6) [312]
12m	Practiced	135 (87.1) [155]	95 (58.3) [163]	-	230 (72.3) [318]
	Did not practice	16 (10.3) [155]	68 (41.7) [163]	-	84 (26.4) [318]

Notes: *LEC = Life Events Checklist. EPQ = Eysenck Personality Questionnaire, neuroticism subscale, SS-FF = Social Support Family and Friends, SS-W = Social Support Work, CAT = Concrete and Abstract Thinking Questionnaire.*

© 2026 Wild G et al. *JAMA Network Open*.

eTable 4: Rates of participants achieving completion, compliance and minimum dose per condition

Adherence Measure	iCT-R (N/%)	Edu (N/%)
**Completion** (all 6 modules)	142 (72.8)	153 (77.6)
**Minimum Compliance**	182 (93.0)	184 (93.4)
**Minimum Dose**	162 (83.1)	167 (84.7)

Notes: Completion is defined as completion of all 6 modules. Minimum compliance is defined as Modules 1 and 2 or Module 6 for both interventions. Minimum dose includes Modules 3 and 4 (iCT-R), and Modules 3 and 6 (Edu). Module descriptions are provided in eTable 2.

© 2026 Wild G et al. *JAMA Network Open*.

eTable 5: Rates of completion per module and time spent on modules by intervention condition

Module	iCT-R (n=195)	Edu (n=197)
**Module 1**	**It Matters What you Focus On: Helpful and Unhelpful Attention**	**Stress**
Participants completed, N (%)	188 (96.4)	187 (94.9)
Fully completed, N (%)	179 (95.2)	187 (100.0)
Partially completed, N (%)	9 (4.8)	0
Median duration, mins	38.4	6.0
IQR, mins	17.8 – 64.2	2.9 – 10.8
**Module 2**	**Get Out of Your Head with Helpful Thinking**	**Sleep**
Participants completed, N (%)	178 (91.3)	184 (93.4)
Fully completed, N (%)	166 (93.3)	179 (97.3)
Partially completed, N (%)	12 (6.7)	5 (2.7)
Median duration, mins	37.4	8.0
IQR, mins	24.6 – 140.8	3.4 – 14.4
**Module 3**	**Habits and Dwelling: How to Change Them**	**Depression**
Participants completed, N (%)	170 (87.1)	177 (89.8)
Fully completed, N (%)	161 (94.7)	172 (97.2)
Partially completed, N (%)	9 (5.3)	5 (2.8)
Median duration, mins	19.2	9.5
IQR, mins	10.4 – 28.9	3.2 – 23.3
**Module 4**	**Dealing with Unwanted Memories: Then versus Now**	**Anger**
Participants completed, N (%)	169 (86.7)	170 (86.3)
Fully completed, N (%)	158 (93.5)	168 (98.8)
Partially completed, N (%)	11 (6.5)	2 (1.2)
Median duration, mins	23.0	6.2
IQR, mins	13.0 – 47.9	2.4 – 13.7
**Module 5**	**Transforming Worries and Improving Performance**	**Mindfulness**
Participants completed, N (%)	158 (81.0)	172 (87.3)
Fully completed, N (%)	147 (93.0)	169 (98.3)
Partially completed, N (%)	11 (7.0)	3 (1.7)
Median duration, mins	15.3	5.7
IQR, mins	8.4 – 31.5	1.7 – 10.4
**Module 6**	**Beating Stress and Trauma: My Blueprint**	**PTSD**
Participants completed, N (%)	163 (83.6)	169 (85.8)
Fully completed, N (%)	160 (98.2)	166 (98.2)

© 2026 Wild G et al. *JAMA Network Open*.

Partially completed, N (%)	3 (1.8)	3 (1.8)
Median duration, mins	13.9	5.6
IQR, mins	6.9 – 25.8	2.2 – 12.9

Notes: iCT-R = Internet-delivered cognitive training in resilience; Edu = Psychoeducation. Percentages for completion rates are calculated from those who initiated each module. IQR = Interquartile range.

© 2026 Wild G et al. *JAMA Network Open*.

eAppendix 3. Sensitivity, CACE and Mediation Analyses

Sensitivity analyses were conducted to examine the robustness of the primary analysis results by analysing data with missing cases included and examining potential covariates predictive of missing data. One variable (hospitalisation) showed a significant predictive relationship of missingness. Participants who reported at baseline being hospitalised in the past six months were more likely to have missing data on the SCID at 1-year (OR= 3.24, 95%CI [1.53, 6.88], p=.002). As a sensitivity analysis, this variable was included as an additional covariate in the primary analysis model. This did not alter the substantive results of the model.

Three participants with baseline diagnoses of PTSD and MDD had been randomly allocated to iCT-R or psychoeducation intervention groups and were included in all primary analyses. Additional sensitivity analyses examined rates of diagnoses at 1-year follow-up with these three participants excluded. Table 6 presents the primary analysis with these baseline cases removed. iCT-R continued to demonstrate significantly lower rates of PTSD and MDD diagnoses at 1-year follow-up compared to psychoeducation (OR=0.23, 95% CI [0.06, 0.84], p=.026). When compared to standard practice, iCT-R demonstrated lower rates of PTSD and MDD at follow-up, approaching but not achieving statistical significance (OR=3.71, 95% CI [0.97, 14.30], p=.056).

© 2026 Wild G et al. *JAMA Network Open*.

eTable 6. Sensitivity Analysis. Primary analysis with the three baseline clinical cases removed

Measure	Time	N meeting criteria			Adjusted difference [95%CI], *p* value		
		iCT-R	Edu	Wait	iCT-R vs Wait	Edu vs Wait	iCT-R vs Edu
SCID PTSD and/or Depression	Pre	0	0	0			
	Post	3	3	4	2.23 [0.39, 12.81], .365	1.63 [0.35, 7.69], .535	0.73 [0.12, 4.53], .736
	12m	3	14	10	3.71 [0.97, 14.30], .056	0.84 [0.34, 2.08], .706	0.23 [0.06, 0.84], .026

Notes. Model specification was identical to the Primary analysis. Adjusted differences and confidence intervals given on the log odds ratio scale.

We analysed outcomes for participants who exceeded the minimal compliance threshold and participants who completed core modules. Minimal compliance was defined as completion of either modules 1 and 2 or module 6 in either intervention. For iCT-R, core module completion was defined as completing module 3 (Habits and Dwelling: How to change them) and module 4 (Dealing with unwanted memories: Then vs Now). For the psychoeducation intervention, core module completion was defined as completing module 3 (Depression) and module 6 (PTSD). Participants in iCT-R who exceeded the minimum compliance threshold were significantly less likely to develop PTSD and MDD by 1 year follow-up compared to participants who exceeded the minimum compliance threshold for psychoeducation, (OR=0.19, 95% CI [0.05, 0.77], p=.020). Similarly, participants who completed core modules in iCT-R were significantly less likely to be diagnosed with PTSD or MDD at 1 year follow-up compared to psychoeducation, (OR=0.15, 95% CI [0.03, 0.79], p=.024).

© 2026 Wild G et al. *JAMA Network Open*.

eTable 7. Sensitivity Analyses (Minimal compliance, and core module completion): Odds of meeting criteria for PTSD and MDD at follow-up

	Measure	Time	N meeting criteria		Adjusted difference [95%CI], *p* value
			iCT-R	Edu	iCT-R vs Edu
Participants exceeding minimum compliance threshold	SCID PTSD and/or Depression	Pre	1	1	
		Post	3	3	1.06 [0.19, 5.75], .949
		12m	3	13	0.19 [0.05, 0.77], .020
Participants completing core modules	SCID PTSD and/or Depression	Pre	1	1	
		Post	3	2	1.84 [0.27, 12.43], .529
		12m	2	12	0.15 [0.03, 0.79], .024

Notes. Model specification was identical to the Primary analysis. Adjusted differences given on the odds ratio scale.

© 2026 Wild G et al. *JAMA Network Open*.

eTable 8. Mediation of 12-month Scores on the PCL-5 and PHQ-9

Outcome	Mediator		Total Effect Adjusted Difference (SE) [95%CI]	Total Effect p	Direct Effect Adjusted Difference (SE) [95%CI]	Direct Effect p	Indirect Effect Adjusted Difference (SE) [95%CI]	Indirect Effect p	% Mediated
PCL-5	RIQ Dwelling	iCT vs Wait	2.55 (0.93) [0.72, 4.38]	.006	2.22 (0.93) [0.39, 4.05]	.017	-0.08 (0.25) [-0.57, 0.42]	.756	-
		Edu vs Wait	0.52 (0.92) [-1.29, 2.33]	.573	0.49 (0.91) [-1.30, 2.28]	.589	0.12 (0.25) [-0.37, 0.61]	.622	-
	Wagnild	iCT vs Wait	2.52 (0.94) [0.66, 4.38]	.008	2.24 (0.98) [0.33, 4.16]	.022	0.05 (0.06) [-0.07, 0.16]	.433	-
		Edu vs Wait	0.46 (0.94) [-1.38, 2.30]	.623	0.09 (0.95) [-1.78, 1.97]	.922	-0.02 (0.04) [-0.09, 0.05]	.566	-
PHQ-9	RIQ Dwelling	iCT vs Wait	1.00 (0.40) [0.23, 1.78]	.012	1.23 (0.40) [0.43, 2.02]	.003	-0.02 (0.07) [-0.16, 0.12]	.756	-
		Edu vs Wait	0.42 (0.39) [-0.35, 1.19]	.286	0.62 (0.4) [-0.16, 1.39]	.121	0.03 (0.07) [-0.10, 0.17]	.624	-
	Wagnild	iCT vs Wait	0.96 (0.40) [0.18, 1.75]	.016	1.33 (0.41) [0.52, 2.13]	.001	0.10 (0.06) [-0.03, 0.22]	.134	-
		Edu vs Wait	0.36 (0.40) [-0.41, 1.14]	.359	0.46 (0.40) [-0.33, 1.25]	.255	-0.04 (0.06) [-0.16, 0.07]	.463	-

Notes. The mediator was the posttreatment/postwait score on the process variable listed, and the outcome was the 12-month score on the symptom measure (PCL-5 or PHQ-9). Coefficients estimated using linear mixed effects models, with total, direct, and indirect effects calculated following Baron and Kenny (First, 2015). Models included baseline scores on the mediator, PCL-5, and PHQ-9, and gender as fixed covariates, with a random effect of participants nested within universities.

Mediation analyses tested whether group differences in PTSD and depression symptom improvement at 12 months were mediated by disorder-related rumination and resilience scores postintervention. There were no significant indirect effects (all ps>0.05). eTable3 shows the results of mediation analyses.

© 2026 Wild G et al. *JAMA Network Open*.
