# Supplementary material for: Cognitive Resilience Training to Prevent PTSD and Major Depressive Disorder in Paramedic Recruits: A Randomized Clinical Trial
Source: JAMA Netw Open. 2026 Feb 9;9(2):e2557241. doi: 10.1001/jamanetworkopen.2025.57241 (PMC12887744; doi:10.1001/jamanetworkopen.2025.57241)
Supplement: Supplement 3. — Data Sharing Statement [file jamanetwopen-e2557241-s003.pdf]

# Data Sharing Statement

Wild. Cognitive Resilience Training to Prevent PTSD and Major Depressive Disorder in Paramedic Recruits. *JAMA Netw Open*. Published February 04, 2026.
doi:10.1001/jamanetworkopen.2025.57241

**Data**
**Additional Information:** The name of the trial registry: ISRCTN URL: https://www.isrctn.com/
Trial Registration Number: ISRCTN16493616
**Data available:** No

**Additional Information**
**Explanation for why data not available:** The dataset contains detailed accounts of traumatic events experienced by participants, including incident descriptions that, even when de-identified, could compromise participant anonymity given the specialised nature of paramedic work. Researchers interested in accessing anonymised summary data may contact the corresponding author.
